# Normal Mutation Rate Variants Arise in a Mutator (Mut S) *Escherichia coli* Population

**DOI:** 10.1371/journal.pone.0072963

**Published:** 2013-09-12

**Authors:** María-Carmen Turrientes, Fernando Baquero, Bruce R. Levin, José-Luis Martínez, Aida Ripoll, José-María González-Alba, Raquel Tobes, Marina Manrique, Maria-Rosario Baquero, Mario-José Rodríguez-Domínguez, Rafael Cantón, Juan-Carlos Galán

**Affiliations:** 1 Department of Microbiology, Ramón y Cajal Institute for Health Research, Madrid, Spain; 2 Centro de Investigación Biomedica en Red de Epidemiología y Salud Pública, Carlos III Health Institute, Madrid, Spain; 3 Joint Unit for Research in Antibiotic Resistance and Virulence, Madrid, Spain; 4 Department of Biology, Emory University, Atlanta Georgia, United States of America; 5 Department of Microbial Biotechnology, Centro Nacional de Biotecnología, Madrid, Spain; 6 Research Department, Era7 Bioinformatics, Granada, Spain; 7 Faculty of Health Sciences, Alfonso X El Sabio University, Madrid, Spain; University of Massachusetts Medical School, United States of America

## Abstract

The rate at which mutations are generated is central to the pace of evolution. Although this rate is remarkably similar amongst all cellular organisms, bacterial strains with mutation rates 100 fold greater than the modal rates of their species are commonly isolated from natural sources and emerge in experimental populations. Theoretical studies postulate and empirical studies teort the hypotheses that these “mutator” strains evolved in response to selection for elevated rates of generation of inherited variation that enable bacteria to adapt to novel and/or rapidly changing environments. Less clear are the conditions under which selection will favor reductions in mutation rates. Declines in rates of mutation for established populations of mutator bacteria are not anticipated if such changes are attributed to the costs of augmented rates of generation of deleterious mutations. Here we report experimental evidence of evolution towards reduced mutation rates in a clinical isolate of *Escherichia coli* with an hyper-mutable phenotype due a deletion in a mismatch repair gene, (Δ*mutS*). The emergence in a Δ*mutS* background of variants with mutation rates approaching those of the normal rates of strains carrying wild-type MutS was associated with increase in fitness with respect to ancestral strain. We postulate that such an increase in fitness could be attributed to the emergence of mechanisms driving a permanent “aerobic style of life”, the negative consequence of this behavior being regulated by the evolution of mechanisms protecting the cell against increased endogenous oxidative radicals involved in DNA damage, and thus reducing mutation rate. Gene expression assays and full sequencing of evolved mutator and normo-mutable variants supports the hypothesis. In conclusion, we postulate that the observed reductions in mutation rate are coincidental to, rather than, the selective force responsible for this evolution.

## Introduction

The modal mutation rate (μ) is remarkably similar among cellular organisms with DNA genomes, typically μ = 5×10^−9^ per base pair per cell division [1], [2]. In *Escherichia coli* the most accurate available values indicate that spontaneous mutations occur at a rate of 1×10^−3^ per genome and generation [3]. The value 8.9×10^−11^ per base pair per cell division was inferred from whole-genome synonymous substitutions in a long-term evolution experiment with *E. coli* [4], Although they are rare among laboratory strains [5], [6], [7], a proportion of bacteria from natural sources, including clinical isolates, can have mutation rates two or more orders of magnitude greater than this modal value [8], [9]. It is generally assumed, demonstrable with mathematical and computer simulation models [10] and supported experimentally [11], that these “mutator” strains are favored in situations where bacteria confront continually changing physical and/or biotic environments. The reason for selection of mutators is that an elevated mutation rate makes more likely that a bacterium will produce adaptive variants. Moreover, if all else is equal, populations with higher mutation rates are anticipated to adapt to novel environments more rapidly than those with lower mutations [12]. Observations, made in long-term *in-vitro* evolution experiments, are consistent with this interpretation of the conditions under which selection will favor mutators [13]. What is the downside of having a high mutation rate? If the modal rate of mutation for normo-mutable *E. coli* is some two orders of magnitude less than that of mutators, but these variants are infrequently found in nature, there must be conditions where bacteria with elevated mutation rates are selected-against. What are those conditions? A standard answer is that in addition to being more likely to generate adaptive mutations than normo-mutable strains, mutators are also more likely to generate deleterious mutations which, in the absence of recombination, will accumulate a phenomenon known as Muller's ratchet [14], [15]. This interpretation is supported by extreme bottleneck experiments, where *E. coli* populations are maintained by the sequential growth and transfer of single colonies. Not only do deleterious mutations and reduced fitness arise in mutator lineages under these conditions but some of these populations actually die out [16].

It would seem that a different situation would exist in a sustained population of bacteria with a high mutation rate that is confronted with only modest bottlenecks, where mutations are more likely to be deleterious than adaptive. The reason for this is that if the sole cost of a high mutation rate is due to the generation of deleterious mutations, that fitness burden is going to be small. This can be seen with a thought experiment. Consider the extreme where the likelihood of generating a lethal mutation by a mutator strain is 1000 times greater than that of an otherwise isogenic strain with a normal mutation rate, say 10^−3^ and 10^−6^ per cell per generation, respectively. In other words, the fitness cost of the mutator population relative to the non-mutator would be in the order of 0.001, a burden too low to be measured experimentally.

Consequently, unless the population could be maintained under serial passages for an unrealistic amount of time, a decline in mutation rate would not be anticipated in experimental populations that were initially dominated by mutator strains. This is particularly so because of periodic selection [17], [18], [19] and the bottlenecks associated with transmission between host or habitats, genetic drift purging arising mutants even when they are somewhat beneficial (see Figures S1 and S2, and File S1). Although populations with lower mutation rates and thereby marginally higher fitness will arise and start to increase in frequency, in any given population they are likely to be lost when that population goes through a bottleneck [20]. However, in a seemingly short period of time, M.J. McDonald and colleagues [21] observed declines in the rates of mutation in experimental populations initiated with a high mutation rate strain of the yeast *Saccharomyces cerevisiae*. The reason(s) for the ascent of the lower mutation rates variants in this study have yet to be elucidated. How might low-mutation rates be selected?

Particular mutation rates are not by themselves selectable phenotypes. Strains with high mutation rates are selected only because of them have a greater possibility of acquiring adaptive mutations in genes unrelated with the hyper-mutable phenotype (second-order selection) [22]. We might consider if the emergence of normo-mutable variants within a population of mutators is also of a similar indirect type, as these variants are similarly selected by the association with favorable traits. However, the possibility of finding adaptive mutations is higher for mutators, particularly considering their original quantitative dominance. Consequently, we hypothesize that mutators could evolve a type of advantageous trait that indirectly converts them to nearly normo-mutable strains.

In order to evaluate such a hypothesis, we followed the changes in mutation rate in long-term (1,500 generations) experimental populations of a mutator strain (ECU24) of *E. coli*. Our experimental system was designed to reflect a worse case for the evolution of reduced mutation rates: (i) the elevated mutation rate of the evolving strain was due to an 8 bp deletion in the mismatch repair gene, *mutS*, (ii) the recently isolated urinary tract infection *E. coli* bearing this defective mutator gene would be adapting to laboratory culture and thereby maintained under conditions where elevated mutation rates would be advantageous [12], and (iii) the populations were maintained by serial passage, and thus subject to bottlenecks that would purge rare variants with lower mutation rates. Despite these stringent conditions, we were able to consistently detect variants that retained the original *mutS* deletion but had mutation rates at near modal levels, more than 35 times less than their mutator ancestor. Gene expression and full-sequencing studies indicate that these variants acquired heritable mechanisms to compensate for defects in mismatch repair. We postulate that the decline in mutation rate is coincidental to the acquisition by the mutator population of these increasing-fitness traits, as those reducing DNA-damaging reactive-oxygen species.

## Materials and Methods

### Serial passages and sequential determination of mutational phenotypes

The pathogenic ECU24 *Escherichia coli* strain showing a mutator phenotype was isolated from the urine of a hospitalized patient in Madrid, Spain. The original ECU24 strain with high mutation rate phenotype was submitted to serial passages in Luria-Bertani (LB) Broth during 180 consecutive days (∼1,500 generations approximately). Every day, the culture was diluted 1∶100 in fresh medium (50 µL in 5 mL), and incubated at 37°C under aerobic agitation (200 rev./min). Each two-three days, loop samples were streaked on LB agar plates and three colonies of similar size were chosen for determination of the mutation frequencies (*f*) using rifampicin as selection marker [23].

### Characterization of the ancestor *E.coli* mutator strain

The genes more commonly involved in *E. coli* mutator phenotype (*mutS*, *mutL*, *mutH*, *uvrD*, *mutT* and *mutY*) were amplified from the *E.coli* MG1655 strain using primers and PCR conditions previously described [23]. All reactions were performed in a PTC-100 thermocycler (MJ Research, Inc. Reno, Ne.) using *Taq-gold* (Applied-Biosystems, Branchburg, New Jersey USA). The amplified products were purified using Qiagen columns (QIAGEN GmbH, Hilden, Germany), following the recommendations of the manufacturer. The amplified products of *mutS* (2,727 bp), *mutL* (2,083 bp) *mutH* (832 bp), *uvrD* (2,317 bp), *mutT* (579 bp) and *mutY* (1,130 bp) were cloned in the plasmid pGEMt-easy and transformed into a DH5α *E. coli* strain. Recombinant clones were selected in plates containing ampicillin (50 µg/mL), Xgal (100 µg/mL), and IPTG (40 µg/mL). The clones containing the expected insert were twice re-checked and finally the inserts were sequenced to confirm the absence of mutations when compared with the wild-type strain. The hybrid plasmids carrying each one of the mentioned genes were transformed into the original mutator strain. Mutation frequencies (*f*) were determined for all strains carrying these constructions, according to a previously described protocol [23].

### Estimation of the mutation rate (*μ*) in ancestor *E.coli* mutator strain and evolved variants using different loci

In our work, determination of mutation rates (*μ*) was based on mutation at a single locus, *rpoB* (rifampicin-resistant mutants). In order to exclude artificial results using only one locus, *μ* was also estimated for *rpsL* (streptomycin-resistant mutants) and *gyrA* (nalidixic-resistant mutants) loci. Mutation rates were determined by a modified Luria-Delbrück fluctuation test [24]. Briefly, 100 µL of 20 overnight cultures were independently mixed in soft agar and plated in LB agar plates containing the antibiotic selector (100 µg/mL of rifampicin, or 200 µg/mL of streptomycin, or 20 µg/mL of nalidixic acid). 100 µL of a 10^−6^ dilution from five overnight cultures were used to estimate the total viable count in drug-free LB plates. The Ma-Sandri-Sarkar (MSS)-maximum likelihood method, and the MSS-algorithm [25] were applied in the estimation of the number of mutants.

### PFGE of *E. coli* ECU24 colonies obtained along the serial passages experiment

In order to discard problems of bacterial contamination along the experiment, pulsed-field gel electrophoresis (PFGE) comparative assays were performed on four mutator and four normo-mutable colonies isolated at the different periods (ancestor, 75^th^, 102^nd^, 150^th^, 180^th^) along the experimental evolution assay. Bacterial DNA was prepared as described previously [26] and XbaI (Roche GmbH, Mannheim, Germany) was used as restriction enzyme. Digested DNA was separated in a CHEF-DRIII (Bio-Rad, La Jolla, Ca.) and the conditions were as follows: 14°C, 6 V/cm, 10 to 40 s, 27 h.

### RNA expression profiling

Transcription profiles of three independent mutator clones, selected among those with lower mutation frequency values (*f*≈4–6×10^−7^), and three others from the normo-mutable variants (*f*≈4–6×10^−8^), all of them recovered in a late period (passage 151^st^), were compared with that of the ancestral mutator strain. Total RNA was extracted from the ancestral strain and two of its evolved mutators (mutation rate 3.41×10^−8^, lower than the original mutator strain) and normo-mutable variants (mutation rate 2.8×10^−9^) from the 151^st^ passage grown at mid-logarithmic phase in Minimal Davies broth supplemented with glucose 1 g/L, using Rneasy mini kit (Qiagen GmbH, Germany). Three colonies belonging to the evolved mutator or the normo-mutable variant population were chosen, and gene expression was tested in two independent replicate experiments. The quality and quantity of extracted RNA was estimated in a ND-100NanoDrop spectrophotometer (Thermo Fisher Scientific, Inc. USA). RNA expression profiling was performed using the Affymetrix Genechip® technology (Progenika Biopharma SA, Spain) following the protocol recommended by the manufacturer.

### Sequencing, assembly, annotation and genome comparison

Full genomes from the ancestor (t_0_) strain, as well as from two evolved mutator strains (t_151_, t_180_), and two normo-mutable variants coexisting in the same cultures (t_151_, t_180_) were sequenced using illumina paired end reads with insert size of around 450 bp. The 5 genomes were sequenced using illumina paired end reads with insert size of around 450 bp. The coverage obtained was 197× for t_0_ ancestor genome, 125× for t_151_ evolved mutator genome, 175× for t_180_ evolved mutator genome, 177× for t_151_ evolved normo-mutable variant genome, and 155× for t_180_ evolved normo-mutable variant genome. The reads were analyzed and pre-processed with PrinSeq [27] trimming the right end where the quality was lower than Q30 and filtering out those reads containing Ns. A *de novo* assembly was carried out using Velvet [28]. The assemblies were done using high values for k parameter to reduce the possibility of misassembles. The prediction of genes and the functional annotation was carried out using BG7 [29]. A *de novo* assembly was carried out using Velvet [28]. The four evolved genomes were aligned against the genome of the parent strain t_0_ to analyze the differences. We used the MAUVE tool “Move Contigs” [30] for the alignment and ordering of the contigs with respect to the reference genome. A specific in house java program for analyzing the differences between the two aligned genomes was used for detecting all the differences of type deletions, insertions and SNPs. The differences detected were analyzed in the BG7 annotation context considering their allocation in coding regions or intergenic regions. The synonymous/non synonymous amino acid changes produced by each SNP were also exhaustively analyzed. SNPs provoking amino acid changes in conserved genes were selected and analyzed in detail.

### Estimated fitness based on relative growth rate (RGR)

To determine the growth rate (α), eight colonies with a mutator phenotype and eight from normo-mutable variants obtained along the serial passages were selected, as well as the clones tested for gene-expression. Five independent replicates of each evolved variant were inoculated into 400 µL of Minimal Davis Broth (Becton Dickinson Co., USA) supplemented with glucose 1 g/L (final density of ∼10^5^ cfu/mL) and incubated at 37°C. Optical density over time (580 nm) was measured by using a Labsystems Bioscreen C Analyzer (ThermoLab Systems, Finland). The RGR of each of the evolved variants was calculated as the ratio of the growth rates (α) in evolved variants with respect to those of the ancestral strain (mean of 10 independent replicates): α_evolved variant_/α_ancestral strain_. Identical procedure was applied to determine the comparative fitness of the variants of the ancestral strain resulting from the introduction of the plasmids pGEMT*nei*, pGEMT*sodB*, and pGEMT*katG*. In this experiment, the RGR was obtained in 5 independent essays for each one of three clones derived from each variant strain (and the ancestor).

### Statistical methods

Continuous variables were described by the mean and standard deviation. Categorical variables were described by relative and absolute frequencies. The Kruskal-Wallis and Mann-Whitney tests were used to compare mutation frequencies in the populations. Signification level used was 0.05, bilateral contrast. The SPSS statistical package for Windows, version 16, was used for statistical analysis.

## Results

### The *E. coli* ECU24 strain and the experimental evolution assay

We performed serial passages (until reaching 1,500 generations) with the mutator *E. coli* ECU24 strain (genome length 5224560 bp, this work) isolated from a human urinary tract infection, in an unstructured environment (LB broth). The rifampicin-resistance mutation rate (*μ*) of this strain was 1.87×10^−7^ (with a confidence interval, CI, at 95% ranging 1.52×10^−7^–2.23×10^−7^) ∼35-fold higher than the modal value for *E. coli*. The mutator phenotype of the ancestor ECU24 strain was ascertained with the loci *rpoB* (rifampicin resistance), *rpsL* (streptomycin resistance) and *gyrA* (nalidixic acid resistance) as markers. Mutation rates were1.87×10^−7^ (with a CI at 95% (CI95%) ranging 1.52×10^−7^–2.23×10^−7^) for *rpoB*, 4.42×10^−7^ for *rpsL* (CI 3.40×10^−7^–5.51×10^−7^) and 2.71×10^−7^ for *gyrA* (CI 2.29×10^−7^–3.15×10^−7^) in the ancestor strain.

The genes usually responsible for a mutator phenotype in *E. coli*, namely *mutS*, *mutL*, *mutH*, *uvrD*, *mutT* and *mutY* were amplified and sequenced in *E. coli* ECU24. The only mutation found was an 8 bp deletion (ACGCCCAT) 19 bp downstream the *mutS* gene initiation codon, giving rise to a frameshift in the open reading frame. Complementation with a wild-type *mutS* gene reduced *μ* until 6.35×10^−9^ (confidence interval at 95% ranging 4.79×10^−9^–8.07×10^−9^) ∼30-fold lower than the original clinical strain, confirming that the cause of hyper-mutable phenotype in *E. coli* ECU24 is a defect in MutS. This value was retained as characterizing the normal mutation rate for a possible normo-mutable *E. coli* ECU24. The possible involvement of other genes commonly implicated in the mutator phenotype was ruled out (see text S1 in File S2).

### Screening and verification of normo-mutable variants

Screening for potential reductions in mutation rate was based on the sequential determination of mutation frequencies (*f*) of colonies obtained from serial passages. In this work, the term “mutation frequency” means the frequency of rifampicin-resistant mutant colonies/ml with regard to the total viable colony count/ml of the same culture. We keep the classic standard “mutation frequency” designation, knowing that the real one is “mutant frequency”. As this marker (*f*) is not always faithfully reproducible, the range of variability in *f* was defined for the original mutator strain and its isogenic non-mutator *mutS*-complemented derivative using 80 colonies from each mutational phenotype. Non-overlapping ranges were obtained defining the limits of high and normo- mutation frequency variants from *f* = 2.20×10^−6^ to 7.25×10^−8^ and *f* = 6.38×10^−8^ to 5.95×10^−9^ respectively. Hence, the breakpoint to discriminate normal and mutator mutational phenotypes was established as *f* = 7.00×10^−8^. During the evolution experiment, *f* was estimated for three independent colonies obtained from cultures every-third/fourth day. In total, mutation frequencies of 186 individual colonies from 62 passages were obtained.

**Figure 1:**
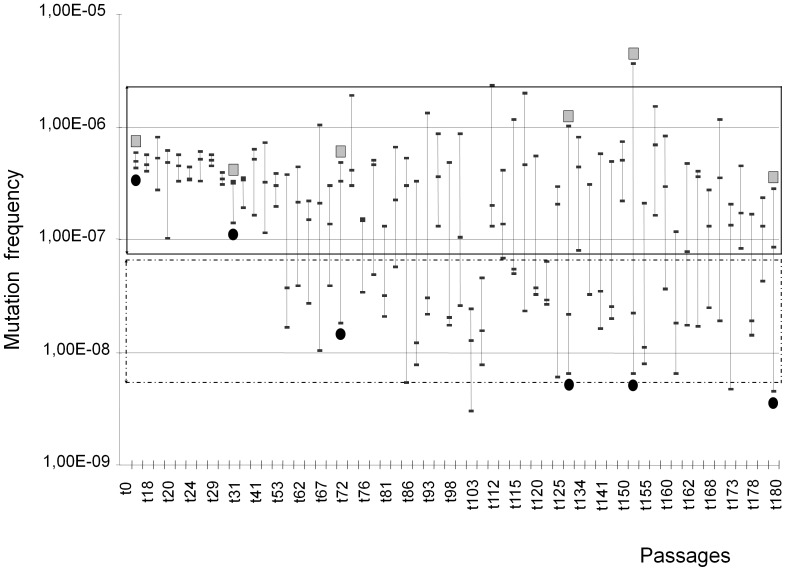
Distribution of mutation frequencies of *E. coli* ECU24 mutator strain along 62 serial passages. In the *y* axis, mutation frequencies; in the *x* axis, numbers of the serial passages in which mutation frequencies were determined (three colonies per passage, 186 in total); note that for clarity not all passages are numbered in the figure. Top box in black continuous line refers to the range of mutation frequencies (80) determined for the original mutator strain; down box in black broken line refers to the range of mutation frequencies determined in the mutator strain after complementation with the wild-type *mutS* gene. Grey squares correspond to colonies with high mutation frequencies where mutation rates were determined (Luria-Delbrück method); black circles, to colonies with low mutation frequencies where mutation rates were obtained.

**Figure 2:**
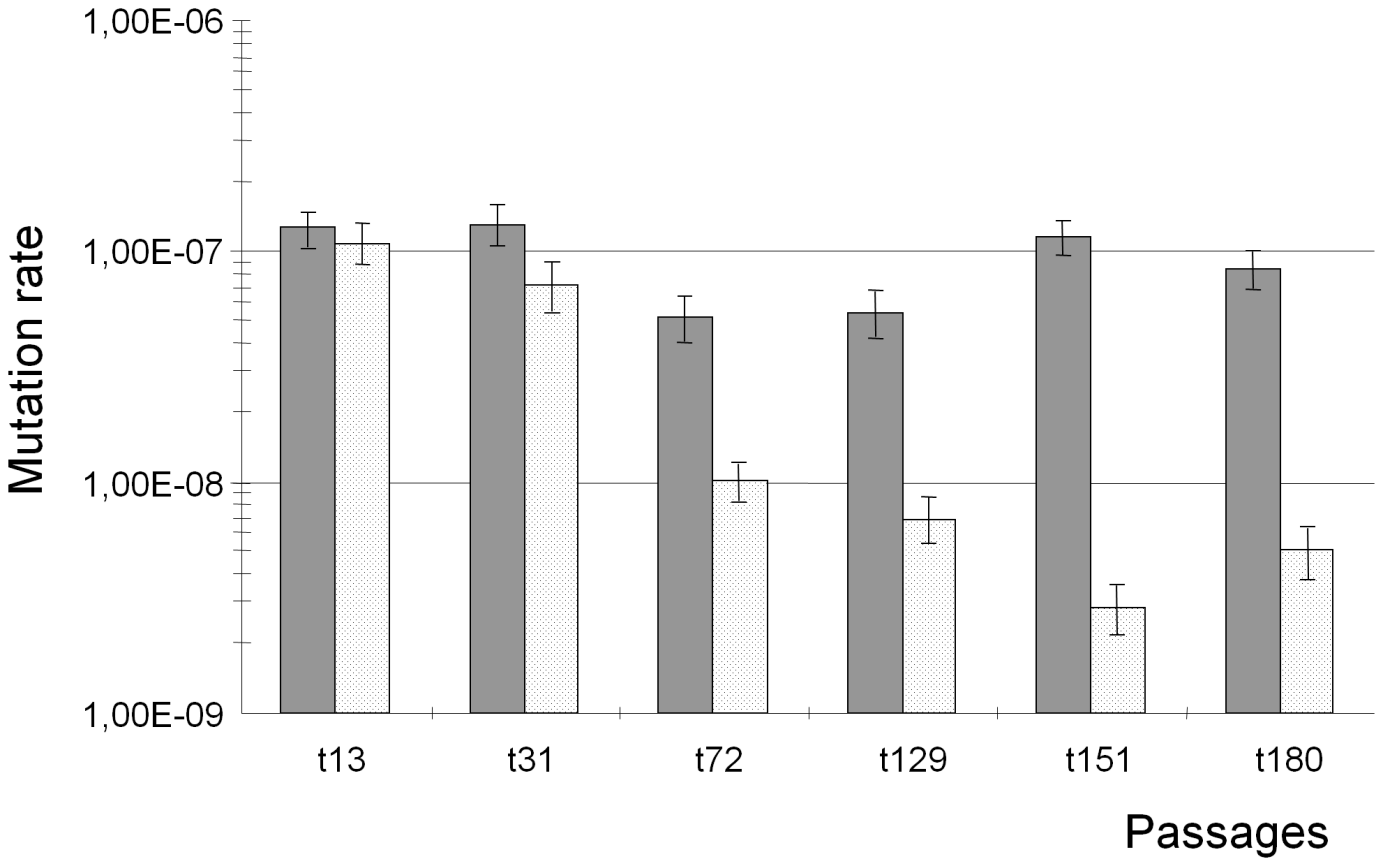
Mutation rates of colonies with high and low mutation frequencies obtained in the same passage, along time. In the *y* axis, mutation rates (Luria-Delbrück method); in the *x* axis, numbers of the serial passages in which mutation rates were determined. Bars in grey correspond to colonies with high mutation frequencies; white dotted bars correspond to colonies with low mutation frequencies.

The distribution of mutation frequencies revealed that it was only after the 53rd passage and until the end of the experiment that passages contained colonies with mutation frequencies compatible with normo-mutable phenotypes (Figure 1). To confirm the normo-mutable nature of these variants, the mutation rates (based on the Luria-Delbrück method) [24], [31] from three variant colonies with normal mutation frequencies obtained at the 72^nd^, 129^th^, and 151^st^ passages, yielded mutation rates respectively of 1.01×10^− the^ (CI95% 8.23×10^−9^–1.21×10^−8^); 6.94×10^−9^ (CI95% 5.42×10^−9^–8.61×10^−9^) and 2.87×10^−9^ (CI95% 2.16×10^−9^–3.64×10^−9^) (Figure 2). Mutation rates of three colonies with mutation frequencies ranging in the mutator phenotype obtained in the same four passages were of 5.19×10^−8^ (CI 95% 4.05×10^−8^–6.43×10^−8^); 5.43×10^−8^ (CI95% 4.17×10^−8^–6.76×10^−8^) and 1.6×10^−7^ (CI 95% 9.65×10^−8^–1.36×10^−7^) respectively (Figure 2). Moreover, the mutation rates of colonies with the mutator or normal mutation phenotype recovered in a late passage (180^th^) was ascertained with the three loci *rpoB* (rifampicin resistance), *rpsL* (streptomycin resistance) and *gyrA* (nalidixic acid resistance) as markers. The corresponding mutation rates for colonies with mutator or normal mutation were 1.41×10^−7^ (CI 95% 1.15×10^−7^–1.70×10^−7^); 3.74×10^−7^ (2.78×10^−7^–4.83×10^−7^); 8.35×10^−8^ (CI 95% 6.80×10^−8^–1.01×10^−7^) and 1.91×10^−8^ (1.30×10^−8^–2.61×10^−8^) for mutator, and 5.08×10^−9^ (3.8×10^−9^–6.49×10^−9^), 4.22×10^−8^ (3.26×10^−8^–5.27×10^−8^); 2.36×10^−9^ (1.91×10^−9^–3.92×10^−9^) and 5,08×10^−9^ (CI95% 3.80×10^−9^–6.49×10^−8^) for normal mutation colonies. These results confirmed the expected outcome concerning maintenance of the normo-mutable and strong-mutator populations along the serial passages and the end of the experiment.

The most striking finding was the documentation of the unexpected possibility of the emergence of normo-mutable variants from a Δ*mutS E. coli* mutator strain, and the coexistence in the same culture of populations with different mutation rates. Most importantly, we proved that all seven normo-mutable variant colonies obtained late in the experiment, in the 146^th^, 150^th^, 151^st^ and 180^th^ passages maintained the altered *mutS* sequence of the ancestor strain, which was identical to that of five evolved mutator colonies from the same passages. All *mutS* genes showed the same 8 bp deletion and the sequence was identical to that of the ancestral sequence. This result shows that the emergence of variants with normal mutation rates was not due to the restoration of the MutS function by changes in the *mutS* sequence. Full genome sequencing confirmed the results obtained along the evolution experiment, and provided additional evidence of the lack of modifications in the genetic neighborhood of *mutS*.

In order to discard problems of bacterial contamination along the experiment, pulsed-field gel electrophoresis (PFGE) comparative assays were performed on four colonies with the mutator phenotype and another four with the normo-mutable phenotype, isolated at the different periods (ancestor, 75^th^, 102^nd^, 150^th^, and 180^th^) along the experimental evolution assay. The PFGE pattern of the original population showed no change in colonies obtained during serial passages, supporting the absence of contamination along the experiment (see Figure S3). Full genome sequencing confirmed the expected high similarity between evolved variants and the ancestor strain, confirming that these variants were derived of the same ancestral clone.

The emergence in serial passages of variants with low-mutation frequencies was repeatable in a simplified (80 passages) confirmatory experiment with six independent evolving replicates derived from the same ancestral mutator population, whose *f* values were estimated along 80 serial passages Colonies with *f* values corresponding to the normo-mutable phenotype (*f* = 6.38×10^−8^ to 5.95×10^−9^) were detected in all six bacterial replicate populations derived from the same ancestral population (see text S2 in File S2).

### Time of emergence of normo-mutable variants

**Figure 3:**
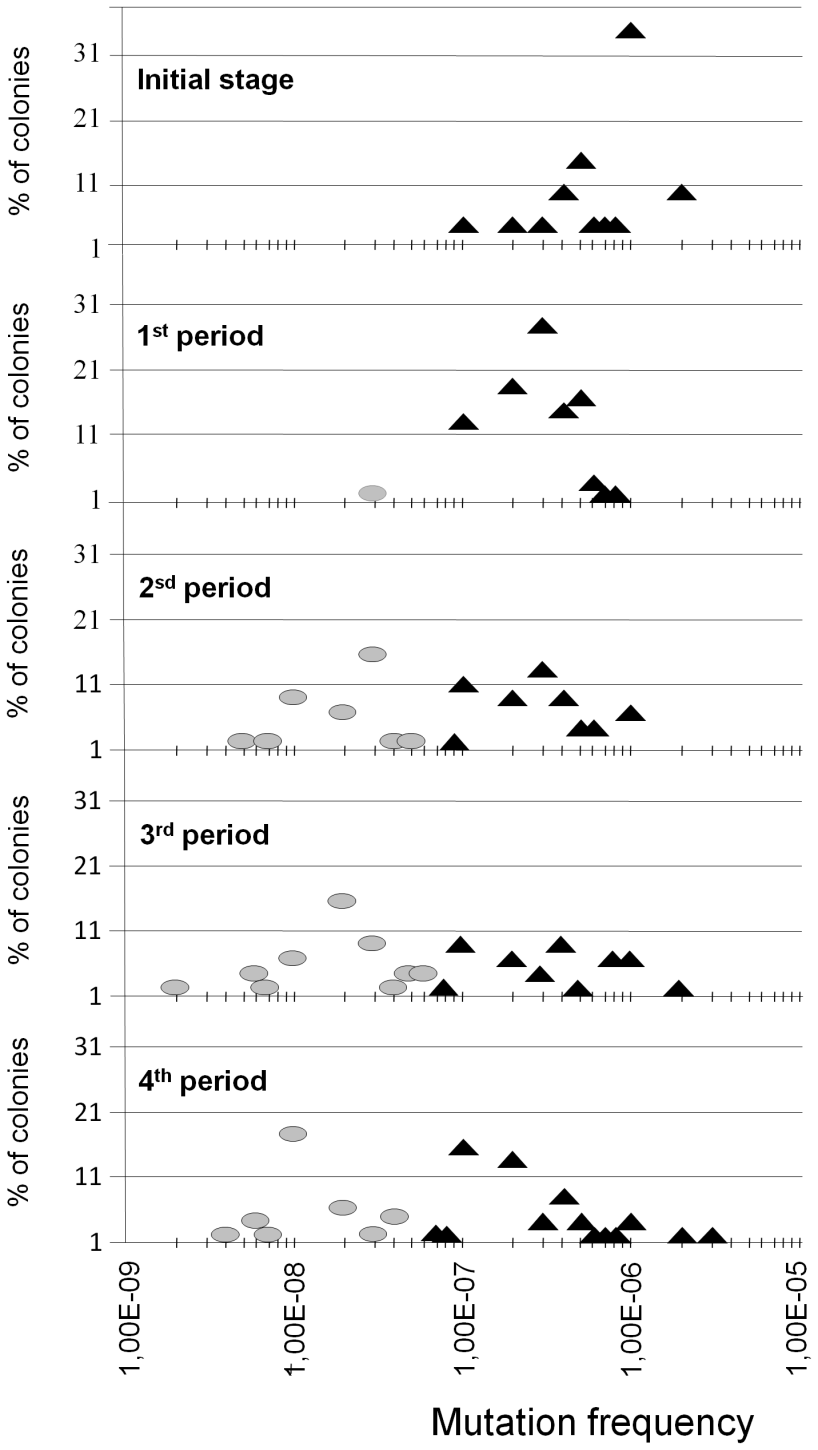
Distribution of the proportion of colonies with high and low mutation frequencies along four successive periods. On the top, distribution of *f* values in 20 colonies of the ancestor strain; below, distributions of *f* values in 45–51 colonies from 15 different passages belonging to each one of the 1^st^ (from 13^th^ to 53^rd^ passage), 2^nd^ (from 58^th^ to 93^rd^ passage), 3^rd^ (from 97^th^ to 138^th^ passage) and 4^th^ (from 141^st^ to 180^th^ passage) periods respectively. Black triangles and grey circles correspond to colonies with high- or low frequencies of mutation respectively.

**Figure 4:**
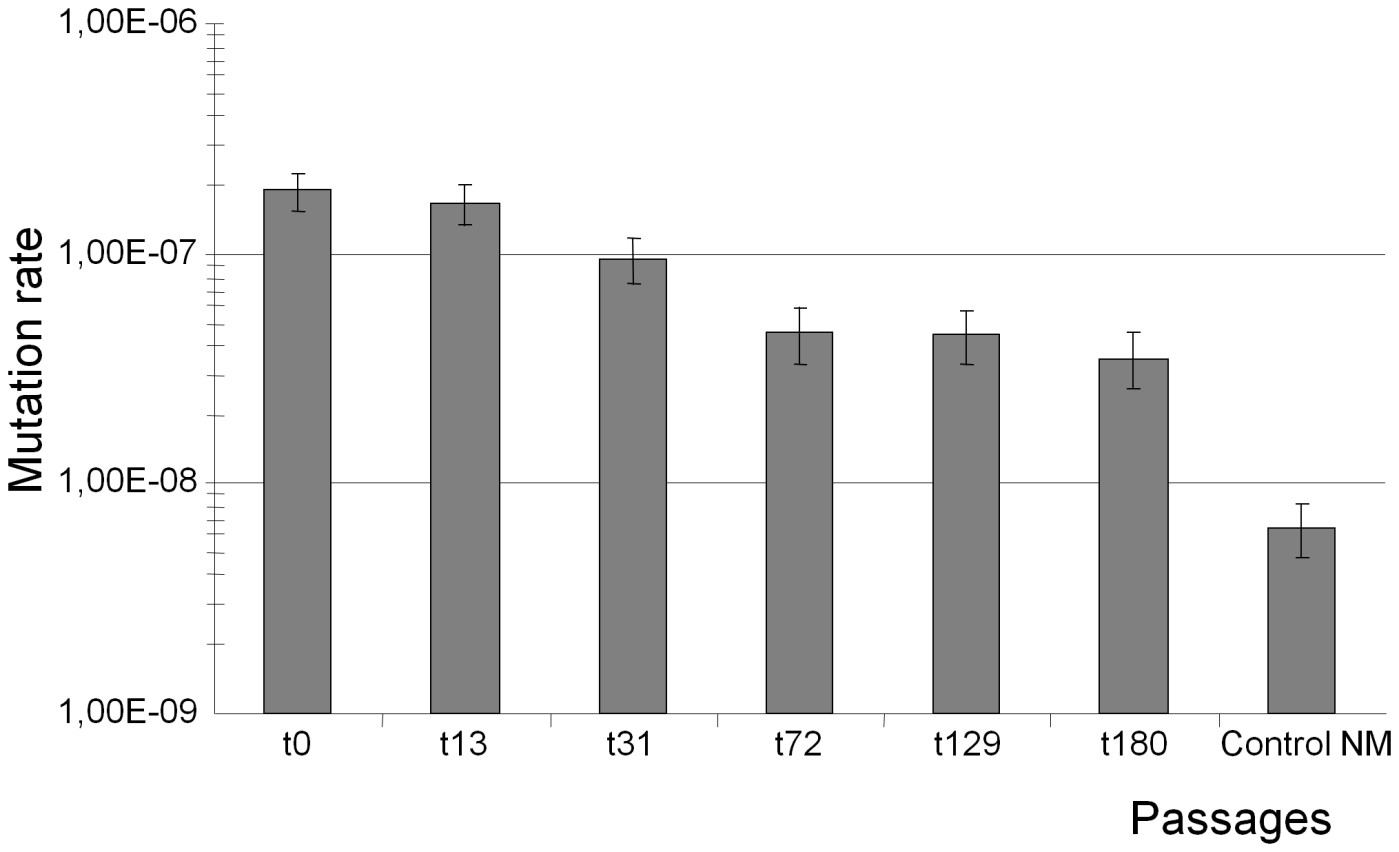
Mutation rates of cultures along the long-term sequential passages experiment. In the *y* axis, mutation rates (Luria-Delbrück method) in the *x* axis, numbers of the serial passages in which mutation rates were determined. Bars reflect the corresponding ranges. The last column on the right side is the mutation rate of the original mutator strain after complementation with the wild-type *mutS* gene, serving as a control for non-mutator mutation rate.

Because of the hyper-mutable nature of the ancestor strain, a continuous generation of different variants is expected to occur during serial passages. What we observed is that such a variation was able to provide clones with normal mutation rates at measurable frequencies. When do normo-mutable variants emerge? According to the rough data in Figure 1, the first colonies within the range of normo-mutation appeared around the 53^rd^ passage (350 generations), but due to the bias of the sampling method, normo-mutable variants might have emerged much earlier. To determine this primary observation, we collected the mutation frequencies of 186 colonies recovered along four arbitrary intervals (1^st^ period, 13^th^ to 53^rd^; 2^nd^ period, 58^th^ to 93^rd^; 3^rd^ period, 97^th^ to 141^st^, and 4^th^ period, 146^th^ to 180^th^ passages). For each interval, 45–51 colonies were obtained (three colonies from 15 individual passages per period; 17 for the last period, i.e., every 2–3 days sampling). The proportion of colonies at each mutation frequency interval was plotted (Figure 3). Statistical analysis of the 45–51 values obtained for each interval indicates that mutation frequencies were significantly modified from the 1^st^ period to either the 2^nd^, 3^rd^, and 4^th^ period (p<0.0001 in all cases), but not among any one of the three last periods. In fact the overall mutation rates obtained for particular cultures were reduced from the 1^st^ to 2^nd^ period, and were maintained stable from the 2^nd^ to the 4^th^ periods (Figure 4). Therefore, a consistent visible emergence of variants with normal mutation frequencies seems to occur after 50 passages (about 400 generations). That suggests that these clones might have a selective advantage to ascend to frequencies where they were detected, and high enough to cross the transmission bottleneck (see text S3 in File S2).

Fitness of normo-mutable and mutator colonies obtained along the experiment was measured as relative growth rates (σ) in relation to original mutator clinical strain (arbitrary assigned as σ = 1.00). During the first ∼400 generations, while the mutator population remained essentially homogeneous in terms of mutation rate, fitness was increased, reaching a mean σ = 1.69. In successive periods, the mean σ values of the mutator-derived colonies were reduced from the optimum 1.69 to 1.46 and 1.54 in the 2^nd^, and 3^rd^ periods, evolving back to the lower level of the ancestral strain (0.96) in the 4^th^ period. Normo-mutable derived colonies had mean fitness values of σ = 1.69 and 1.60 at the 3^rd^, and 4^th^ periods respectively, higher than most of evolved mutator colonies (see Figure S4). However, a fraction of evolved mutator colonies retained a high fitness, explaining the coexistence with evolved normo-mutators at the end of the experiment.

Full genome sequencing of the ancestor and evolved mutators and normo-mutable variants coexisting in the same tubes allowed us to perform a phylogenetic reconstruction (based on 150 concatenated genes with non-synonymous mutations) which confirms again the co-existence of two different evolutionary lineages, each one of them containing both mutators and normo-mutable variants (data non-shown).

### Differences in gene expression among evolved normo-mutable and mutator evolved variants

The global gene expression in colonies obtained late in the experiment (151^st^ passage) was compared with the original mutator strain (see text S4 in File S2). The number of up-regulated genes consistently found among the colonies with high mutation rates and normo-mutable variants was 18 and 62 respectively (see Figure S5); the inverse relation (p<0.001) was observed for the down-regulated or non-expressed genes, with 186 genes in the mutator and 68 genes in the normo-mutable variant population (see Figure S6).

Compared with the ancestor strain, genes which were more transcribed (>1 log_2_ difference) in the normo-mutable evolved variants than in the evolved hyper-mutator strains included *katG* and *sodA*, clearly involved in the ROS detoxification. No significant differences were found in expression of 78 genes potentially involved in mutagenesis, DNA repair, or response to ROS species, as *ada*, *ahpC-F*, *alkA-B*, *dam*, *dcm*, *dinB-G*, *dna*A-*B-C-D-E-G-N-Q-X*, *dps*, *exo*, *fnr*, *fur*, *glyQ-S*, *hns*, *katE*, *lexA*, *mfd*, *miaA*, *mutH-L-M-S-T-Y*, *ndh*, *nfi*, *nfo*, *nth*, *nuo* (operon), *ogt*, *oxyR*, *phrB*, *pnp*, *polA-B*, *ratA-C*, *recA-B-C-D-F-G-J-N-O-R-T*, *rpoS*, *ruv*A-B-C, *sbcB-D*, *sodC*, *ssb*, *topA*, *umuC-D*, *ung*, *uvrA-B-C-D*, *vsr*, or *xthA*. *nei* is the only gene significantly over-expressed in both normo-mutable and evolved hyper-mutable variants. Interestingly, *sodB* was significantly hyper-expressed in the evolved mutator strains, which had increased fitness over the ancestor strain. KatG and SodA, with increased transcription in our normo-mutable variants, favor the elimination of endogenous oxidative radicals involved in DNA damage [32], [33], [34], [35]. It has already been described that the over-expression of genes involved in the intracellular reduction of ROS decreases the mutation rate [32], [36], [37]. The growth fitness of *E. coli* depends on the rate they generate ROS, and a reduction in ROS might increase bacterial fitness [36], [38], [39] so that an increase in the expression of ROS-scavenging genes is a prerequisite for increases in rate of growth [37].

### Over-expression of cloned ROS-scavenging genes in a plasmid (pGEMt-easy) construction reduces mutation rate and increases fitness in the ancestor strain

The genes *katG* (hyper-expressed in evolved normo-mutable strains), *sodB* (hyper-expressed in evolved mutators), and *nei* (hyperexpressed in both evolved normo-mutable and mutators) were cloned into the plasmid pGEMt-easy to assess its influences in mutation rates and fitness. The original mutator strain was independently transformed with pGEMt-*katG* pGEMt-*sodB*, pGEMt-*nei* plasmids, resulting in increased gene dosages. Over-expression of all three genes consistently reduced the μ of the original mutator strain from 18.7×10^−8^ (CI: 14.2–21×10^−8^) to 4.98×10^−8^ (CI: 4.01–6.04×10^−8^) for *katG*, 2.02×10^−8^ (CI: 1.43–2.68×10^−8^) for *sodB*, and 4.26×10^−8^ for *nei* (CI: 2.9–5.78×10^−8^) genes. As expected, over-expression was much less effective in reducing mutation rates in evolved variants where expression of ROS scavenging genes was already up-regulated (see text S5 and Table S2 in File S2), indicating the possible role of enhanced protection to super-oxides in the observed evolution of mutation rates. Nei (endonuclease VIII) belongs to the base excision repair (BER) pathway catalyzing the excision of oxidized pyrimidines, and the effect of *nei* over-expression on the bacterial mutation rate is described here for the first time.

This hypothesis of higher fitness related with enhanced protection from ROS was supported by growth rates experiments, comparing the wild ancestor ECU24 strain and its transformant derivatives with pGEMt-*katG*, pGEMt-*sodB*, and pGEMt-*nei* (see text S6 in File S2). As we have shown previously, these variants had reduced mutation rates. Over-expression of Kat G, SodB, and Nei increased relative growth rates of the ancestor strain by 5%, 35% and 5%, but, as expected, not in the evolved (t_151_) derivatives. These results suggest that increased expression of genes involved in antioxidant defenses might result simultaneously in increased fitness and in reduction of mutation rates.

### The possible ways of restoring normo-mutation in a mutator strain: data from a full genome analysis of evolved normo-mutable variants

Our results indicate that a natural mutator clinical strain might give rise to lower mutation rate variants coexisting in cultures (see text S7 in File S2) in conditions of isolation and in a non-structured environment. According to conventional knowledge, there are three possible ways to reduce the mutation rate in a *mutS* defective variant: i) replacement of the mutated gene for a wild-type allele from a phylogenetically related neighbor donor [40], [41], [42]; ii) back mutation to restore the wild-type allele, and iii) compensatory mutations in additional loci [32]. In our evolution experiment the emergence of low mutation rate variants occurred in the absence of restoration of the defective gene responsible for hyper-mutation, as the *mutS* gene obtained from evolved colonies with high mutation rates maintained the same sequence as the ancestor strain, including the original intragenic deletion. Therefore, the emergence of compensatory mutations neutralizing effects of altered *mutS* is the most plausible explanation.

The genomes of two independent evolved variants (t_151_, t_180_) with a normo-mutable phenotype were fully sequenced and compared with the full genome sequences of the ancestor (t_0_) and two co-existing evolved mutator strains (t_151_, t_180_). All five genomes have the same deletion in *mutS* gene confirming the basic identity of the strain along the process. The high number of transposase genes of the ancestor (81) was maintained in the evolved mutator strains (78 and 79), and in one of the normo-mutable variant strains (74), but was higher in the other one (140 genes). Due to the expected high frequency of transposition events, different inversion-patterns of chromosomal fragments were detected in all four evolved variants, confirming the co-existence of different variants in the same culture tubes (see Figures S7 and S8). Based on the topology of the phylogenetic tree, at least five great recombination events were detected. However, detailed analysis of regions in the neighbourhood of transposition genes did not reveal any possible influence on relevant genes influencing DNA repair or cellular response to oxygen stress.

A careful examination of significant (non-synonymous) nucleotide variation, thus involving amino acid change, was also carried out. In comparison with the ancestor and the two evolved mutator strains, only five genes, three of them involved in oxidative stress protection, including the global regulator *arcA* (aerobic respiration control) and *frdA* (fumarate-reductase) were detected in both normo-mutable evolved strains. The L43P mutation detected in ArcA seems to be associated with a reduction in its functional effects. In fact this regulator represses TCA cycle in micro-aerobiosis, and accordingly to our data TCA cycle was hyper-expressed in normo-mutable variants. Additionally, ArcA also repress Sdh (Succinate-dehydrogenase), SucA-C (oxoglutarate dehydrogenase, SucD (succinyl-CoA synthetase), and CyO (cytochrome-oxydase) and we found that all these genes have increased expression in normo-mutable strains. The mutation in FrdA (R486C) involved in oxidative phosphorylation is difficult to assess is more active under anaerobic conditions [39] out of the already mentioned aerobic style of life of the ArcA mutants. Another gene was changed in only one of the evolved normo-mutable strains (t_180_), a mutation in the gene *atpF* (ATP synthase subunit A), also involved in the oxidative phosphorylation of the respiratory chain. Altered functionality of AtpF is expected to reduce intracellular ATP and the dNTP pool, which might enhance the replicative accuracy of DNA polymerase, thus possibly contributing to a reduction in mutation rates [43]. The net result is that our evolved normo-mutable strains are forced to permanently maintain an aerobic style of life, exposing bacterial organisms to the undesirable consequence of ROS molecules. Routine aerobiosis creates enough oxidative DNA lesions that repair (damaged in our strains) becomes an essential function [39]. ArcA is one of the members of the ArcAB two-component system, which is involved in the resistance of *Escherichia coli* to reactive oxygen stress [44]. We suggest that the reduced activity of ArcA in our evolved normo-mutable strains will increase the intracellular concentration of H_2_O_2_ beyond the threshold required for increased expression of KatG, a highly efficient hydroperoxydase I (catalase), as was documented in our RNA expression profiling experiment. The final outcome is a strong reduction in endogenous ROS-mediated mutagenesis, increase in bacterial fitness and reduction in mutation rates.

The consequences of the mutation found in FrdA (R486C) in normo-mutable evolved variants is difficult to assess, as the encoded fumarate-reductase is more active under anaerobic conditions [39] out of the already mentioned aerobic style of life of the ArcA mutants. The other two genes harbouring mutations in evolved normo-mutable strains coded a “putative uncharacterized protein” (G0D0P9), and a “cytotoxic necrotizing factor type 2” (Q47107), with low probability to influence in a LB culture broth any fitness increase or reduction in mutation rate.

## Discussion

Almost all earlier studies of the *in-vitro* evolution of mutation rates are with competition experiments between laboratory bacterial strains with different rates, μ, submitted to soft or hard-selection [45]. Although these studies provide evidence that if bacteria are subject to frequent, severe (single cell) bottlenecks, mutators would have a disadvantage relative to non-mutators, this is not the case (as in our experimental approach) for sustained populations that are dominated by mutators. In this study, the experimental population was initiated with a clone of *E. coli* that generated a high rate of mutation. As suggested in the Introduction and expanded upon in File S1, if the only cost of a high mutation rate is due to the generation of deleterious mutations then the emergence of strains with normal (lower) mutation rates would not be anticipated to occur during the 1,300 generations of our experiment, or in many more generations. Moreover, since the high mutation rate of the strain of *E coli* used is due to a deletion in a mismatch repair gene, lower rates of mutation could not be generated by reversion with the wild type allele. Finally, since this *E. coli* was recently isolated from the urine of a patient, it was relatively new to laboratory culture, and therefore our experiments were maintained under conditions where the cultures would be adapting to their environment and a high mutation rate would be favored. Be as it may, clones with markedly reduced rates of mutation were recovered.

Why? How? Are the costs of a *mutS* deletion substantially greater than those resulting from errors in DNA replication? While there is evidence that suggests an affirmative answer to this question [5], [46], [47], the mechanisms responsible for this cost have yet to be fully elucidated. Do bacteria have ways to reduce the rate of mutation-inducing DNA replication errors despite a non-functional *mutS*-encoded mismatch repair mechanism? The results of these experiments clearly indicate that this is the case. The strains recovered with lower mutation rates bore the original *mutS* deletion and thereby the reduction in mutation rate can be attributed to mutations elsewhere in the genome, compensatory evolution. Our models show these normal mutation rate variants would require a clear fitness advantage and/or be generated at a high rate to survive and ascend. Such fitness advantage should be higher than the one due to the mere absence of deleterious mutations. Our data suggest that one of these reasons might be the overproduction of ROS-scavenging systems providing fitness advantages.

It is worth noting that, the emergence of low-mutation variants seems to occur only after a fitness increase in the ancestor mutator population, presumably reflecting the acquisition of adaptive changes optimizing its fitness in the new environment, from urine to broth (see Figure S4, and text S3 in File S2). As long as beneficial mutations are occurring in the ancestor population, there is very little chance for the emergence of lower mutation variants [45], [48]. Normo-mutable variants derived from this ancestor retained this level of fitness along the experiment, and more efficiently than mutators. A proportion of mutators coexisting in the same culture with normo-mutable variants retained high fitness, suggesting that mutators are also endowed to increase the possibility of finding compensatory mutations restoring the cost of deleterious mutations [45], [49]. Of course in a culture media submitted to sequential changes in growth phases, the possibility of a polymorphic population is not unexpected [50], [51], [52]. The long-term coexistence of mutators and non-mutators in the same culture flasks was documented by culture along four successive periods (see Figure S9, and text S7 in File S2) and by full-genome sequencing of coexisting evolved strains (see Figures S7 and S8, and text S8 in File S2).

Distribution of mutation frequencies in 20 colonies isolated from the same tube at particular passages along the evolution experiment showed no-detection of normo-mutable strains at t_0_, t_13_, and t_31_; at t_72_ a 25% of the whole population was composed by normo-mutable cells, rising to 35% at t_180_. Competition experiments were carried out in last-passage (t_180_) with *ara*+ variants and *ara*− wild-type cells showing normo-mutable or mutator phenotypes, showing the possibility of full neutrality (see text S9 in File S2). Out of neutrality, evolved polymorphisms might persist over extended periods of time, maintained by metabolic interactions and nutritional trade-offs [53], [54], [55] which might have occurred in our serial passages experiment (see text S9 in File S2).

Presumably there are a number of pathways by which the rate of mutation of strains defective in MutS-encoded mismatch repair can be reduced. We use the term compensatory evolution for this reduction in the rate of mutation, as genetic changes at loci other than *mutS* seem to be responsible for the observed effect. We do not interpret the reduction in the rate of mutation as the selective force responsible for the ascent of these compensatory mutations. In our interpretation the reduction in mutation rate is coincidental to the selective advantage responsible for the ascent of these compensatory mutations, which improved the physiological state of the bacteria for these culture conditions. Resulting changes in the mutators/normo-mutable population structure, more than the accumulation of deleterious mutations influences the decline of mutators, as it has been suggested recently [56]. In an illustrious old precedent of our observation the reduction in hyper-mutability during chemostat growth of a *mutT E. coli* strain was attributed to a presumptive suppressor mutation counteracting the effects of the *mutT* gene [57]. Recently, *mutT* hyper-mutability has been shown to be compensated by *mutY* mutations arising during long-term serial passages experiments [58].

One of the possible “compensatory pathways” we suggest to be considered as responsible for the observed decline in mutation rate involves mechanisms that reduce ROS, that is, superoxides. Removal of superoxides prevents ROS-mediated damage of enzymes with iron-sulphur, impairing bacterial metabolism and consequently growth [44], as well as deleterious effects of stationary-phase induced mutagenesis and death [59], [60], [61], [62], [63], and therefore, over-expression of superoxide-scavenging systems could simultaneously decrease the mutation rate, prevent death in stationary phase and increase bacterial fitness, as was observed in our study. Similar reflections have been recently considered for tumor cells [64].

The results obtained by comparing the full genome sequence of the ancestor strain (genome length 5224560 bp) with that of evolved mutators and evolved normo-mutable strains are highly compatible with such hypothesis. Normo-mutable evolved variants (but not the mutator strains isolated in the same tube, or the ancestor strain), contain a significant mutation in *arcA*, part of a two-component system and an important contributor to the resistance of several Gram negative genera, including *E. coli*, to reactive oxygen species [44], [65], [66]. Decrease in the ArcA functionality also increases the expression of the TCA cycle (as it was shown in our gene expression experiments) and obliges *E. coli* to live permanently in an “aerobic style of life” which results in a higher exposure to H_2_O_2_. ArcA mutation should therefore increase the amount of intracellular ROS, finally resulting in the observed increased expression of *katG*, encoding the highly effective hydroperoxidase I (catalase).

As it was presented above, the expression of *katG* decreases mutation rates, confirming previous studies [67] which suggest that. catalase expression is not solely an emergency response of *E. coli* to environmental oxidative stress, but also that it mediates a homeostatic regulation of the H_2_O_2_ produced by normal aerobic metabolism. This event occurs in our normo-mutable evolved variants as a result of the above mentioned fixation in an “aerobic style of life”.

Our results suggest that a number of self-selectable mechanisms might reduce mutation rates, which might serve in the future as possible targets for interventions aiming to decrease cell mutability [68]. At the same time, they might indicate that a number of “low-level mutators”, frequently found in bacterial organisms isolated from clinical settings [7], [23], [69], [70], could be mutants in the mismatch repair system partially compensated by traits increasing bacterial fitness and reducing mutation rates, as ROS-scavenging systems. A systematic approach to detect anti-mutator genes preventing oxidative DNA damage has already been suggested to identify and characterize oxidation resistance genes [71]. To what extent our observations might be of interest in other areas of research remain to be established; in eukaryotic cells, including experimental *Saccharomyces* populations, and also in malignant tumors, part of these mutator populations tend, to evolve to reduce mutation rates [21], [64]. When human OXR1 gene, protecting from ROS-damage, is introduced in a mutator *mutM mutY E. coli* strain, defective in oxidative DNA repair, a strong reduction in hyper-mutability is observed [72]. Our results indicate that hyper-mutation might paradoxically facilitate such a trend, and consequently it is tempting to suggest that the last evolutionary advantage of high mutation rates might be finding their way back.

## Supporting Information

Figure S1 **Semi-stochastic simulation of the evolution of a higher fitness mutant with in continuous culture with periodic selection.** The maximum growth rates of the N populations are, respectively V_N1_ = 1.0, V_N2_ = 1.1, V_N3_ = 1.2, V_N4_ = 1.3 and V_N5_ = 1.4, the reservoir concentration of the resource is 500 µg/ml and, k = 0.25 and e = 5×10^−7^. The maximum exponential growth rates of the M populations are 0.999 that of the corresponding Ns of that fitness state (a 0.001 fitness cost). The flow rate, w = 0.2 per hour for a generation (doubling time) = 3.47 hours. (A) Changes in the density of cells for five fitness states. The mutation rates to higher fitness states are respectively, 10^−8^, and 10^−6^ per cells per hour for the N and M cell lines. The rate of mutation from M to N is 10^−8^ per cell per hour. (B) Changes in the total cell densities of the N and M populations. 1- No periodic selection. 2- Periodic selection as in Figure S1 (A). 3- Periodic selection as in Figure S1 (A), but no mutation from M to N. 4- Periodic selection as in Figure S1 (A) but with the M->N mutation rate increased to 10^−6^.
(PPT)

Figure S2 **Semi-stochastic simulation of the evolution of higher fitness mutants in continuous culture with periodic bottlenecks.** The maximum growth rates of the N and M populations are, respectively 1.0 and 0.999 (s = 0.001). The reservoir concentration of the resource is 500 µg/ml and, k = 0.25 and e = 5×10^−7^, and the flow rate, w = 0.2 per hour for a generation (doubling time) = 3.47 hours. Changes in the density of the N and M populations, the concentrations of the resource, R, and the ratio of the N/M populations are represented. The probability of a bottleneck, pb = 0.01 per hour and the level of the bottleneck is β = 10^−4^ of the population a. (A) The rate of mutation from M to N is 10^−8^ per cell per hour. (B) The rate of mutation M to N is 10^−6^ per cell per hour.
(PPT)

Figure S3 **PFGE of nine** ***E. coli*** **ECU24 colonies obtained along the serial passages experiment.** Line 1 corresponds to original mutator strain at t_0_; lines 2–3 correspond to colonies with high and normal mutation frequencies in the *second* period (t_76_ passage); lines 4–5 correspond to colonies of the *third* period (t_102_ passage); lines 6–9 correspond to colonies belonging to *fourth* period (t_150_ and t_180_). The PFGE pattern of the original population showed no change in colonies obtained during serial passages, confirming the absence of contamination along the experiment.
(PPT)

Figure S4 **Fitness changes of colonies with high and low mutation frequencies along the serial passages experiment.** Relative growth rates of 66 colonies with high frequency or low frequency of mutation are represented. Black circles correspond to high mutation frequency colonies (including colonies from the ancestral mutator population), and white circles to emerged variants with low frequencies of mutation, compatible with normo-mutation. The unit value corresponds to the average of growth rates in 10 colonies belonging to the ancestral mutator strain. RGR values in the colonies tested along the passages are also the average of 10 replicas.
(PPT)

Figure S5 **Up-regulated genes in evolved (t^151^) normo-mutable and mutator cells.** All genes showing significant (up) differences in the expression level with respect to the ancestor (>0.5 log) were selected following the AffymetrixGenechip® technology. Only genes whose up-regulation was consistently present in replicates from the three colonies belonging to the same group (normo-mutable or mutator) are shown in the figure.
(PPT)

Figure S6 **Down-regulated genes in evolved (t^151^) normo-mutable and mutator cells.** All genes showing significant (down) differences in the expression level with respect to the ancestor (>0.5 log) were selected following the AffymetrixGenechip® technology. Only genes whose down-regulation was consistently present in replicates from the three colonies belonging to the same group (normo-mutable or mutator) are shown in the figure.
(PPT)

Figure S7 **Genome alignment of the two normo-mutable strains t_151_ and t_180_ against the ancestor strain t_0_.** The genomes of t_151_ and t_180_ normo-mutable strains, aligned with progressive MAUVE against the ancestor strain t0, show a global view of their similarity blocks. The different colour blocks interconnected by colour lines represent blocks of sequence similarity between the genomes. Blocks under the alignment black line are fragments that establish their similarity relationship with a reverse orientation of their sequence with respect to the flanking blocks. The red vertical bars limit the different contigs of each genome. The genomes show large blocks of similarity with some insertions/deletions of small fragments (white colour regions into the colour blocks). Some transposition events can be observed as expected considering the high frequency of transposases. Most of the deletions/insertions are in phage regions and in plasmid contigs or are related with different allocations of the transposases active in each genome. Based on MAUVE alignment results the insertions, deletions and SNPs were exhaustively analysed along the genome.
(TIF)

Figure S8 **Genome alignment of the two evolved mutator strains t_151_ and t_180_ against the ancestor strain t_0_.** The genomes of t_151_ and t_180_ evolved mutator strains, aligned with progressive MAUVE against the ancestor strain t0, show a global view of their similarity blocks. The blocks, regions, and bars, and comments correspond to those in Figure S7.
(TIF)

Figure S9 **Distribution of the proportion of colonies with high and low mutation frequencies along four successive periods.** (**A**) At the top, distribution of *f* values in 20 colonies of the ancestor strain; below, distributions of *f* values in 45 colonies from 15 different passages belonging to each one of the 1^st^ (from 13^th^ to 53^rd^ passage), 2^nd^ (from 58^th^ to 93^rd^ passage), 3^rd^ (from 97^th^ to 138^th^ passage) and 4^th^ (from 141^st^ to 180^th^ passage) periods respectively (see text S7 in File S2, is the same as Figure 3; it was reiterated here to facilitate comparisons). (**B**) Distribution of mutation frequencies in 20 colonies isolated from the same tube at particular passages along the evolution experiment. Black triangles and grey circles correspond to colonies with high- or low frequency of mutation respectively. (**C**) Distribution of mutation frequencies in 20 clones derived from single colonies with the highest and lowest *f* values but co-existing in particular passages (13^th^, 72^nd^, 129^th^ and 180^th^). Black triangles and grey circles correspond to colonies with high- or low frequencies of mutation respectively.
(PPT)

File S1 **Supporting text.**
(DOC)

File S2 **Contains supporting text and Table S1 and Table S2.** Table S1. Complementation of the ancestor strain with genes commonly involved in the mutator phenotype. Table S2. Mutation rates of the *Escherichia coli* ECU24 original strain (t_0_), an evolved normo-mutable variant and an evolved mutator variant (both from t_151_ passage) over-expressing Nei, SodB, and KatG.
(DOC)
