# Correction: Normal Mutation Rate Variants Arise in a Mutator (Mut S) *Escherichia coli* Population

**DOI:** 10.1371/annotation/7c826b51-98c5-41d8-a57e-a2836d17857f

**Published:** 2013-09-17

**Authors:** María-Carmen Turrientes, Fernando Baquero, Bruce R. Levin, José-Luis Martínez, Aida Ripoll, José-María González-Alba, Raquel Tobes, Marina Manrique, Maria-Rosario Baquero, Mario-José Rodríguez-Domínguez, Rafael Cantón, Juan-Carlos Galán

There was an error in the Funding statement. The correct version of the Funding statement is available below.

Sources of funding includes Grants LSHM-CT-2005-518152, LSHM-CT-2005-018705,

EvoTAR-282004 and PAR-241476 from the European Union, FIS-PI-12/00567 from Ministry of Economy and Competitiveness of Spain, as well as the US NIH (GM091875, BRL). The funders had no role in study design, data collection and analysis, decision to publish, or preparation of the manuscript. 

