# Supplementary figures and images for: Normal Mutation Rate Variants Arise in a Mutator (Mut S) Escherichia coli Population
Source: PLoS One. 2013 Sep 12;8(9):e72963. doi: 10.1371/journal.pone.0072963 (PMC3771984; doi:10.1371/journal.pone.0072963)

## Slide 1
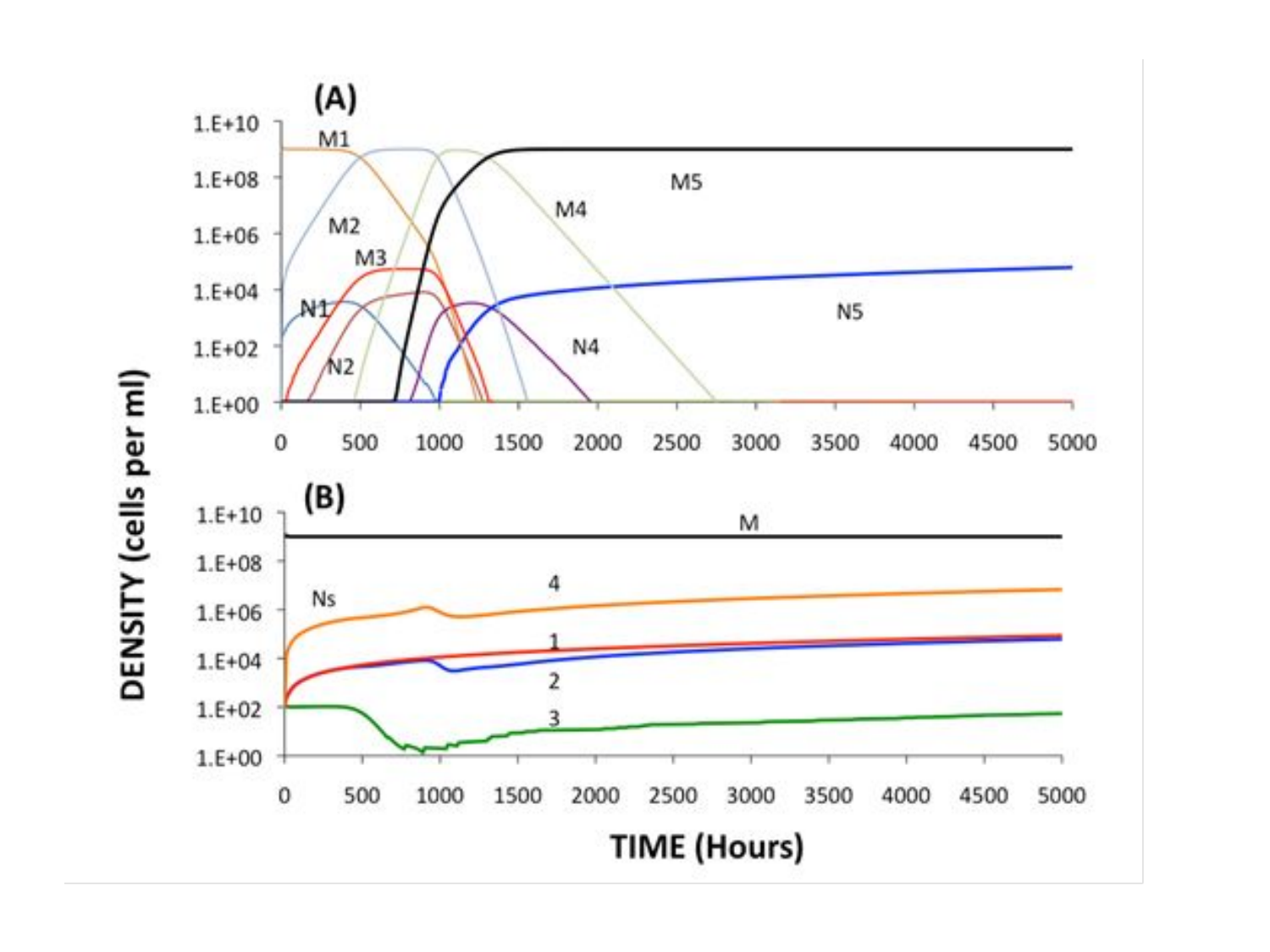

Supplement: Figure S1 — Semi-stochastic simulation of the evolution of a higher fitness mutant with in continuous culture with periodic selection. The maximum growth rates of the N populations are, respectively VN1 = 1.0, VN2 = 1.1, VN3 = 1.2, VN4 = 1.3 and VN5 = 1.4, the reservoir concentration of the resource is 500 µg/ml and, k = 0.25 and e = 5×10^−7^. The maximum exponential growth rates of the M populations are 0.999 that of the corresponding Ns of that fitness state (a 0.001 fitness cost). The flow rate, w = 0.2 per hour for a generation (doubling time) = 3.47 hours. (A) Changes in the density of cells for five fitness states. The mutation rates to higher fitness states are respectively, 10−8, and 10−6 per cells per hour for the N and M cell lines. The rate of mutation from M to N is 10−8 per cell per hour. (B) Changes in the total cell densities of the N and M populations. 1- No periodic selection. 2- Periodic selection as in Figure S1 (A). 3- Periodic selection as in Figure S1 (A), but no mutation from M to N. 4- Periodic selection as in Figure S1 (A) but with the M->N mutation rate increased to 10−6. (PPT) [file pone.0072963.s001.ppt]

## Slide 1
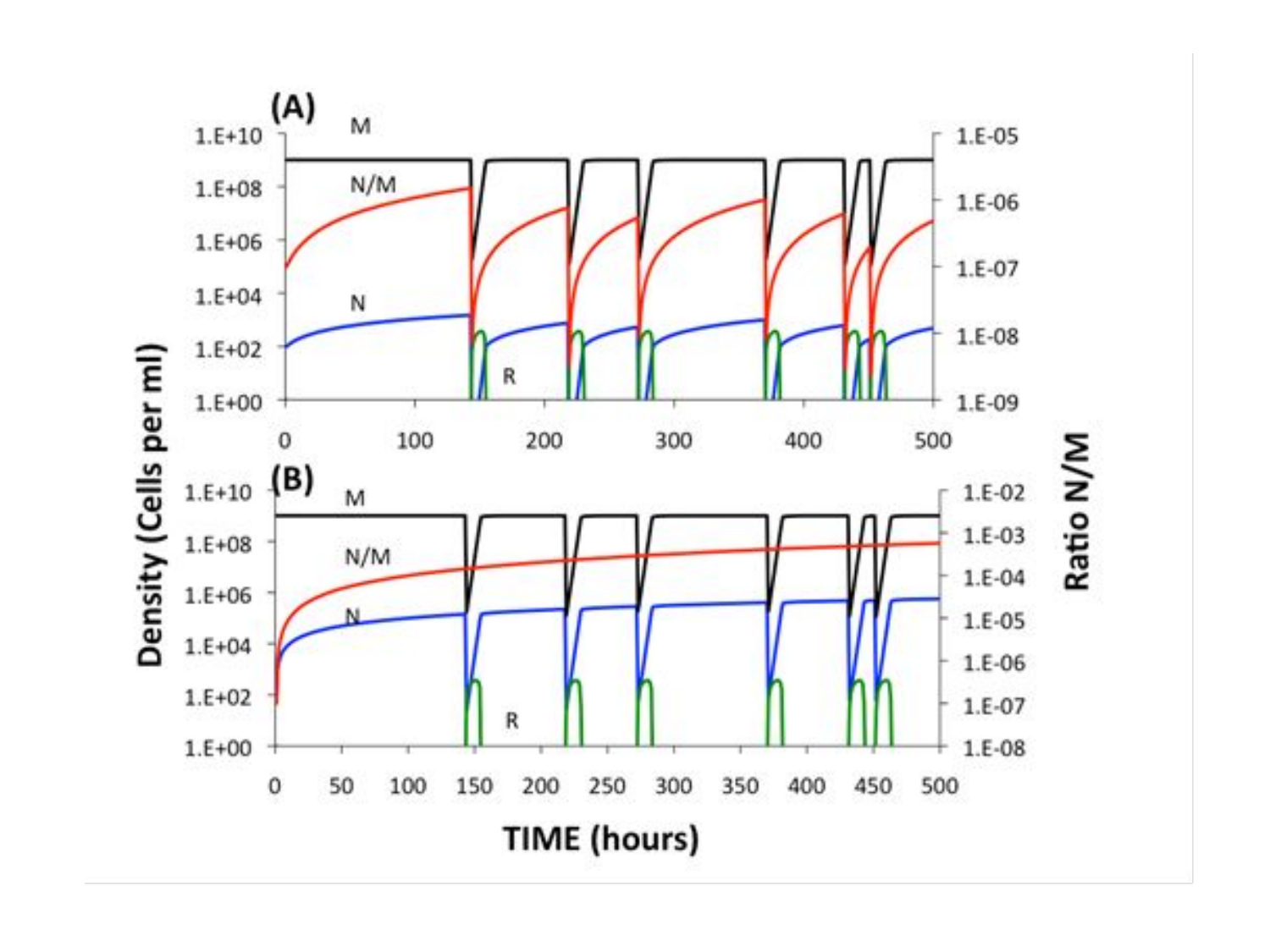

Supplement: Figure S2 — Semi-stochastic simulation of the evolution of higher fitness mutants in continuous culture with periodic bottlenecks. The maximum growth rates of the N and M populations are, respectively 1.0 and 0.999 (s = 0.001). The reservoir concentration of the resource is 500 µg/ml and, k = 0.25 and e = 5×10^−7^, and the flow rate, w = 0.2 per hour for a generation (doubling time) = 3.47 hours. Changes in the density of the N and M populations, the concentrations of the resource, R, and the ratio of the N/M populations are represented. The probability of a bottleneck, pb = 0.01 per hour and the level of the bottleneck is β = 10−4 of the population a. (A) The rate of mutation from M to N is 10−8 per cell per hour. (B) The rate of mutation M to N is 10−6 per cell per hour. (PPT) [file pone.0072963.s002.ppt]

## Slide 1
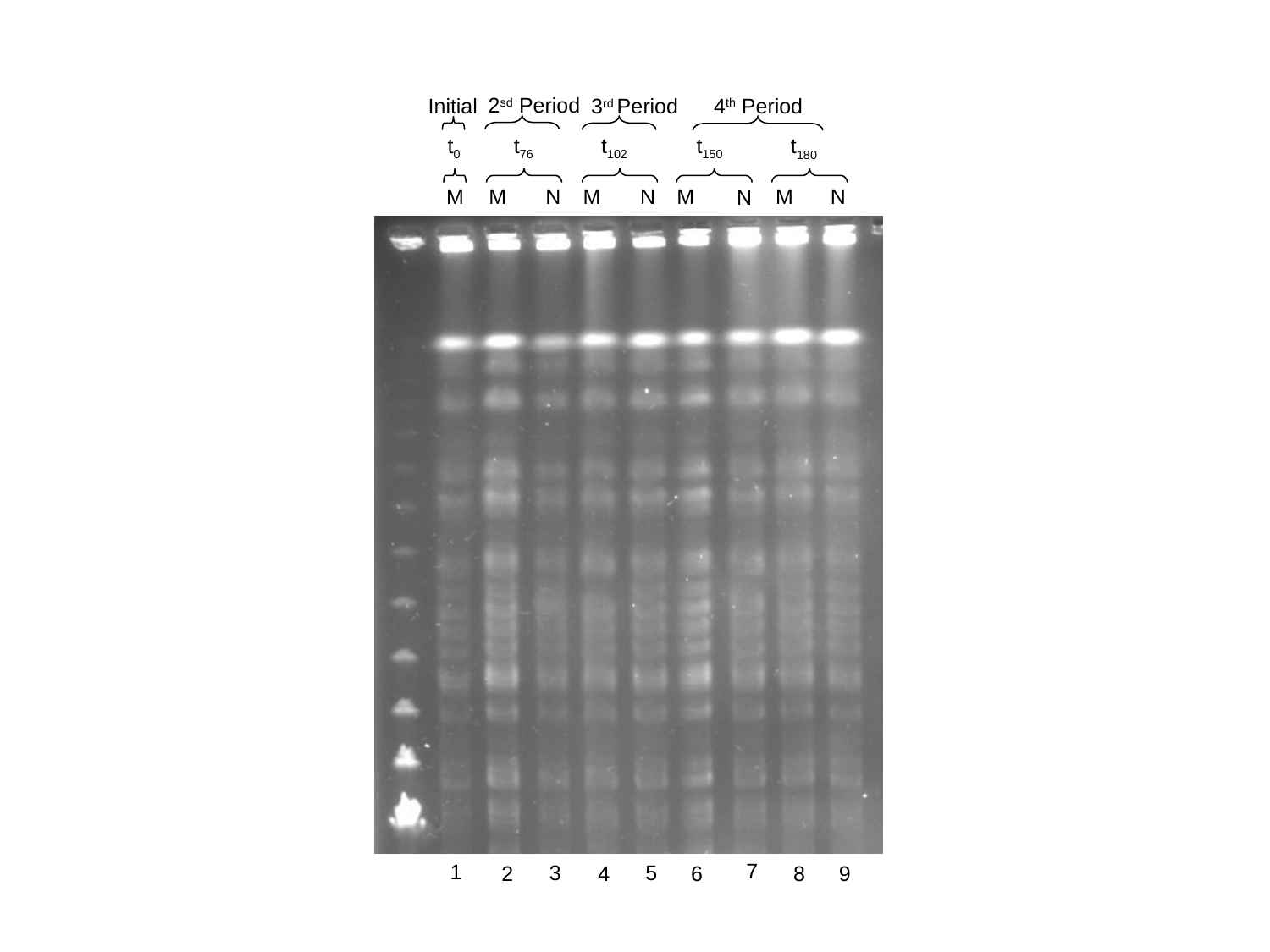

2sd Period
Initial
4th Period
3rd Period
t0
t76
t102
t150
t180
M
M
N
N
M
N
M
M
N
7
1
5
3
2
4
6
8
9

Supplement: Figure S3 — PFGE of nine E. coli ECU24 colonies obtained along the serial passages experiment. Line 1 corresponds to original mutator strain at t0; lines 2–3 correspond to colonies with high and normal mutation frequencies in the second period (t76 passage); lines 4–5 correspond to colonies of the third period (t102 passage); lines 6–9 correspond to colonies belonging to fourth period (t150 and t180). The PFGE pattern of the original population showed no change in colonies obtained during serial passages, confirming the absence of contamination along the experiment. (PPT) [file pone.0072963.s003.ppt]

## Slide 1
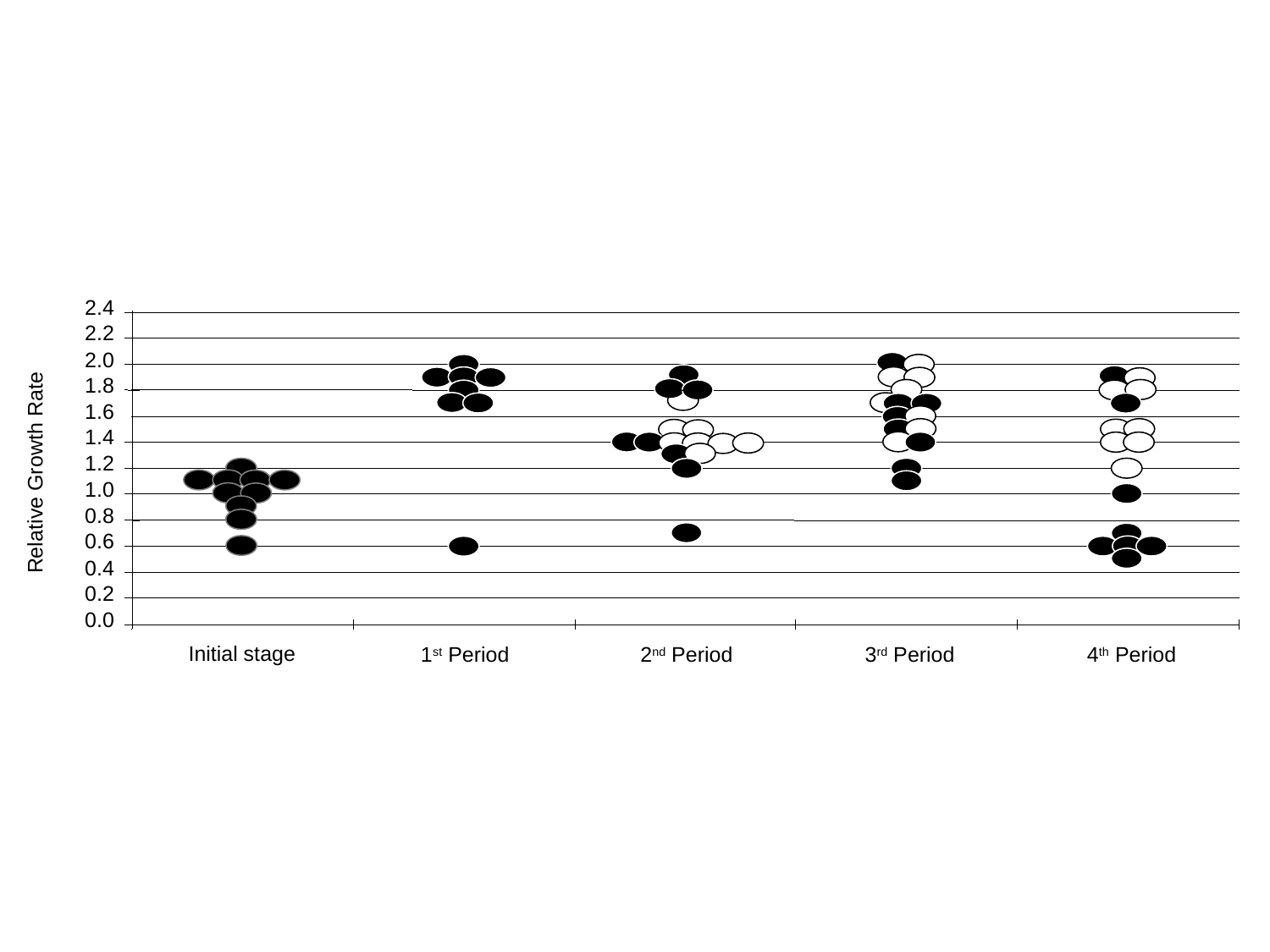

2.4
2.2
2.0
1.8
1.6
1.4
1.2
1.0
0.8
0.6
0.4
0.2
0.0
Initial stage
1st Period
2nd Period
3rd Period
4th Period
Relative Growth Rate

Supplement: Figure S4 — Fitness changes of colonies with high and low mutation frequencies along the serial passages experiment. Relative growth rates of 66 colonies with high frequency or low frequency of mutation are represented. Black circles correspond to high mutation frequency colonies (including colonies from the ancestral mutator population), and white circles to emerged variants with low frequencies of mutation, compatible with normo-mutation. The unit value corresponds to the average of growth rates in 10 colonies belonging to the ancestral mutator strain. RGR values in the colonies tested along the passages are also the average of 10 replicas. (PPT) [file pone.0072963.s004.ppt]

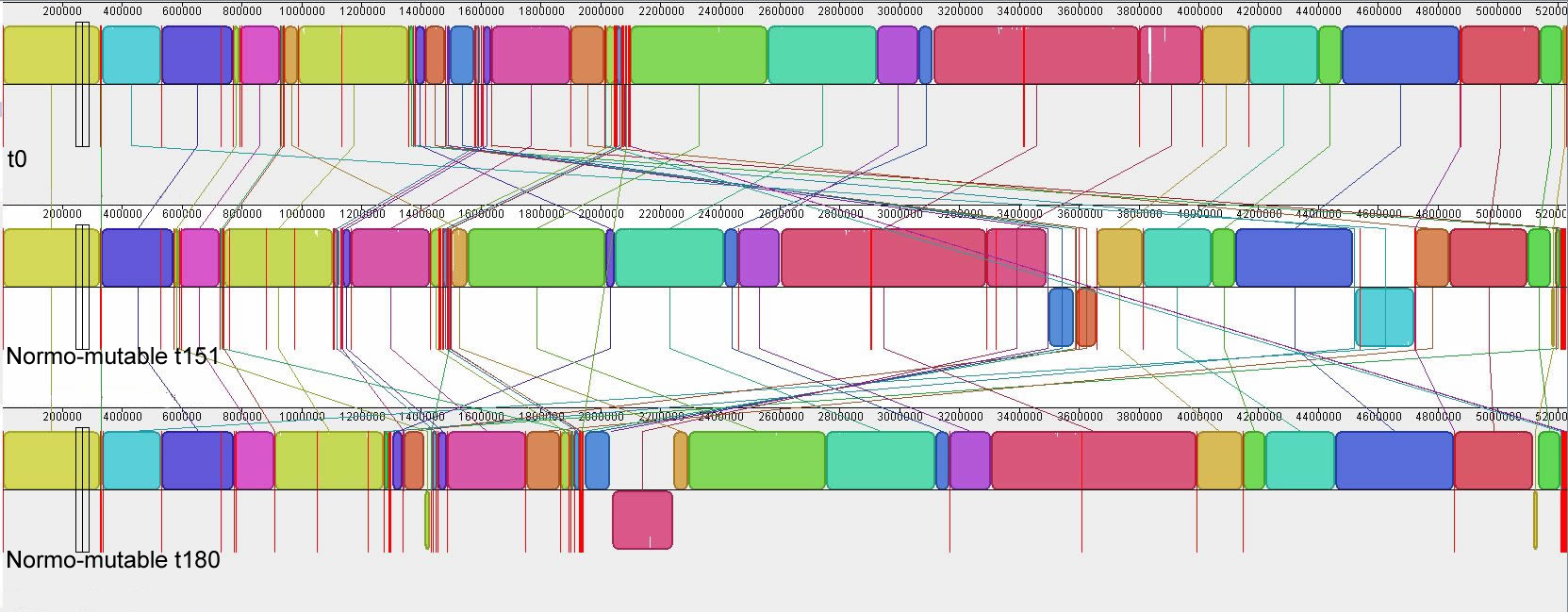

Supplement: Figure S7 — Genome alignment of the two normo-mutable strains t151 and t180 against the ancestor strain t0. The genomes of t151 and t180 normo-mutable strains, aligned with progressive MAUVE against the ancestor strain t0, show a global view of their similarity blocks. The different colour blocks interconnected by colour lines represent blocks of sequence similarity between the genomes. Blocks under the alignment black line are fragments that establish their similarity relationship with a reverse orientation of their sequence with respect to the flanking blocks. The red vertical bars limit the different contigs of each genome. The genomes show large blocks of similarity with some insertions/deletions of small fragments (white colour regions into the colour blocks). Some transposition events can be observed as expected considering the high frequency of transposases. Most of the deletions/insertions are in phage regions and in plasmid contigs or are related with different allocations of the transposases active in each genome. Based on MAUVE alignment results the insertions, deletions and SNPs were exhaustively analysed along the genome. (TIF) [file pone.0072963.s007.tif]

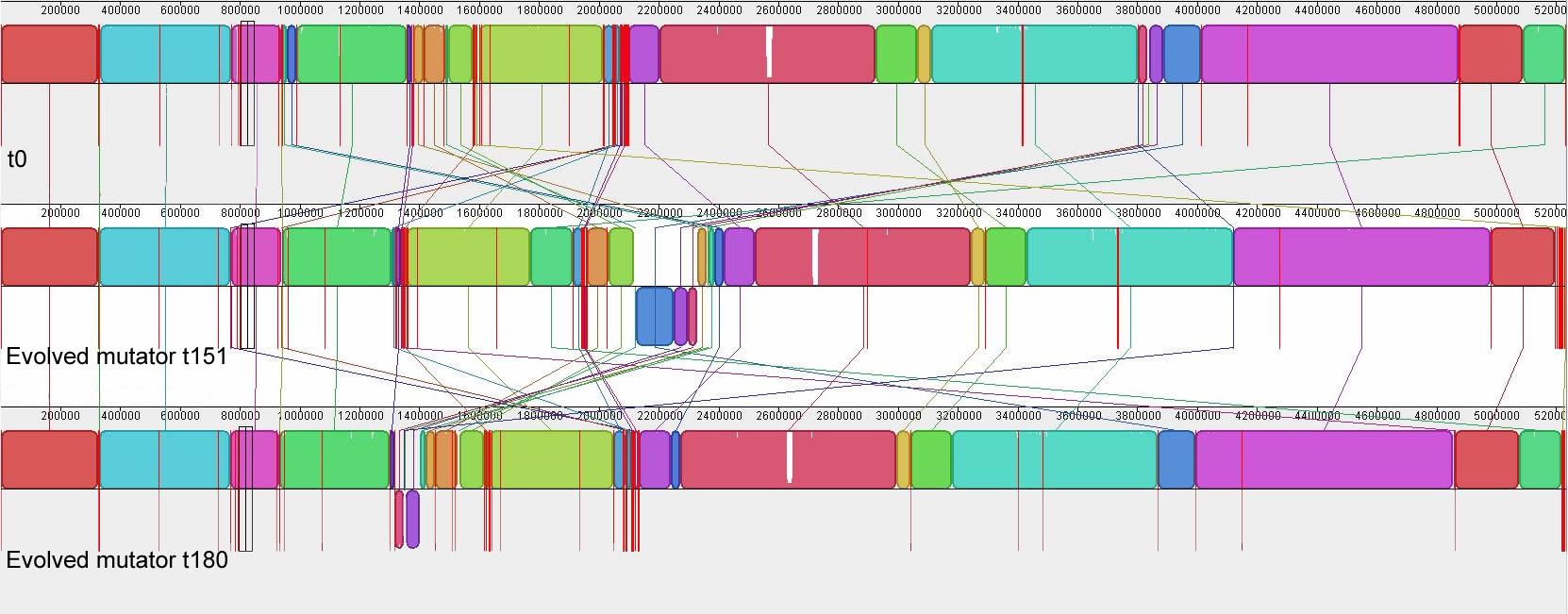

Supplement: Figure S8 — Genome alignment of the two evolved mutator strains t151 and t180 against the ancestor strain t0. The genomes of t151 and t180 evolved mutator strains, aligned with progressive MAUVE against the ancestor strain t0, show a global view of their similarity blocks. The blocks, regions, and bars, and comments correspond to those in Figure S7. (TIF) [file pone.0072963.s008.tif]
