# Supplementary material for: Normal Mutation Rate Variants Arise in a Mutator (Mut S) Escherichia coli Population
Source: PLoS One. 2013 Sep 12;8(9):e72963. doi: 10.1371/journal.pone.0072963 (PMC3771984; doi:10.1371/journal.pone.0072963)
Supplement: Figure S5 — Up-regulated genes in evolved (t151) normo-mutable and mutator cells. All genes showing significant (up) differences in the expression level with respect to the ancestor (>0.5 log) were selected following the AffymetrixGenechip® technology. Only genes whose up-regulation was consistently present in replicates from the three colonies belonging to the same group (normo-mutable or mutator) are shown in the figure. (PPT) [file pone.0072963.s005.ppt]

## Slide 1
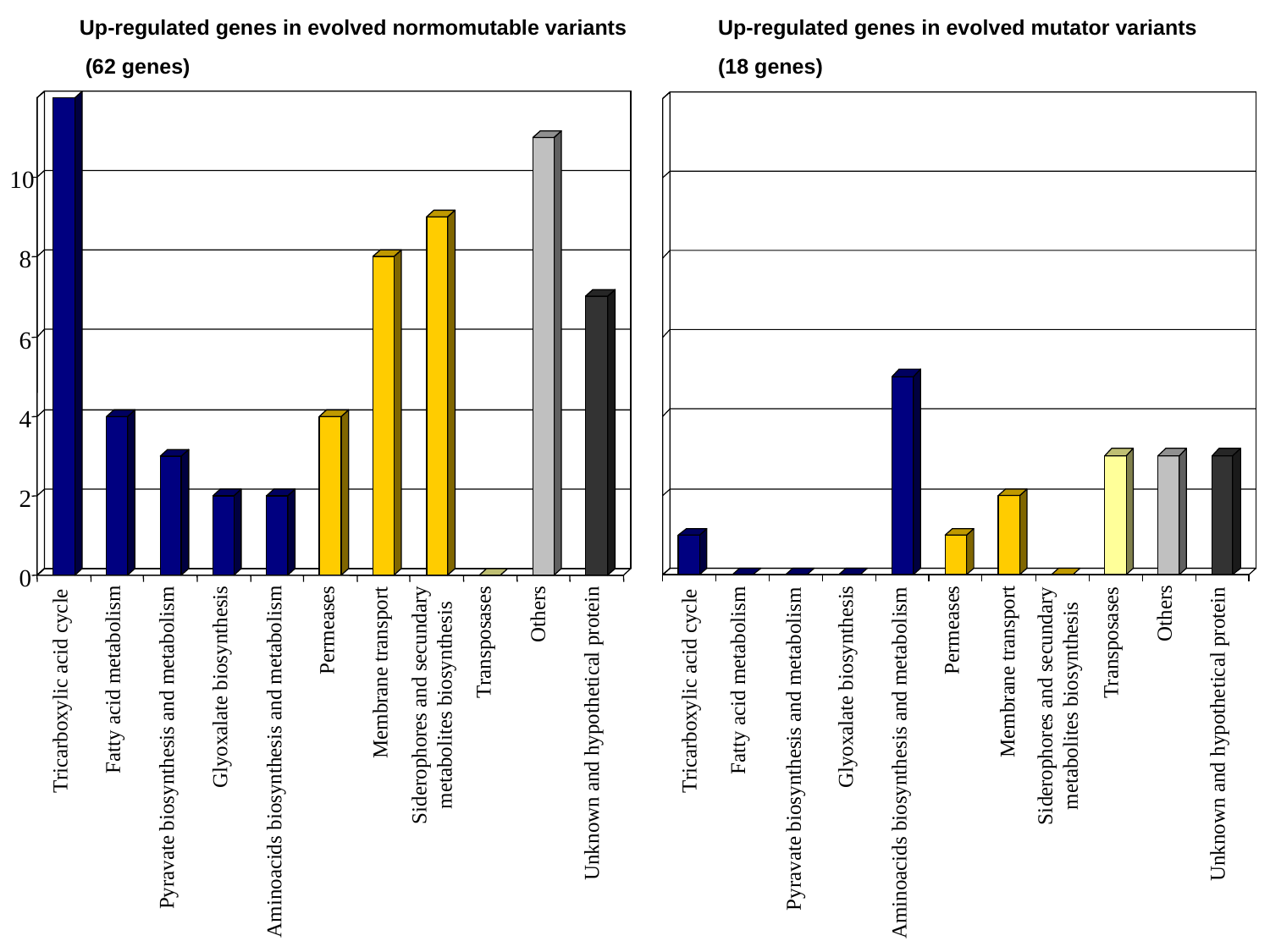

Up-regulated genes in evolved normomutable variants
 (62 genes)
Up-regulated genes in evolved mutator variants
(18 genes)
10
8
6
4
2
0
Others
Permeases
Transposases
Membrane transport
Fatty acid metabolism
 Glyoxalate biosynthesis
Tricarboxylic acid cycle
Siderophores and secundary metabolites biosynthesis
Unknown and hypothetical protein
Pyravate biosynthesis and metabolism
Aminoacids biosynthesis and metabolism
Others
Permeases
Transposases
Tricarboxylic acid cycle
Membrane transport
Fatty acid metabolism
 Glyoxalate biosynthesis
Siderophores and secundary metabolites biosynthesis
Unknown and hypothetical protein
Aminoacids biosynthesis and metabolism
Pyravate biosynthesis and metabolism
