# Supplementary material for: Normal Mutation Rate Variants Arise in a Mutator (Mut S) Escherichia coli Population
Source: PLoS One. 2013 Sep 12;8(9):e72963. doi: 10.1371/journal.pone.0072963 (PMC3771984; doi:10.1371/journal.pone.0072963)
Supplement: Figure S6 — Down-regulated genes in evolved (t151) normo-mutable and mutator cells. All genes showing significant (down) differences in the expression level with respect to the ancestor (>0.5 log) were selected following the AffymetrixGenechip® technology. Only genes whose down-regulation was consistently present in replicates from the three colonies belonging to the same group (normo-mutable or mutator) are shown in the figure. (PPT) [file pone.0072963.s006.ppt]

## Slide 1
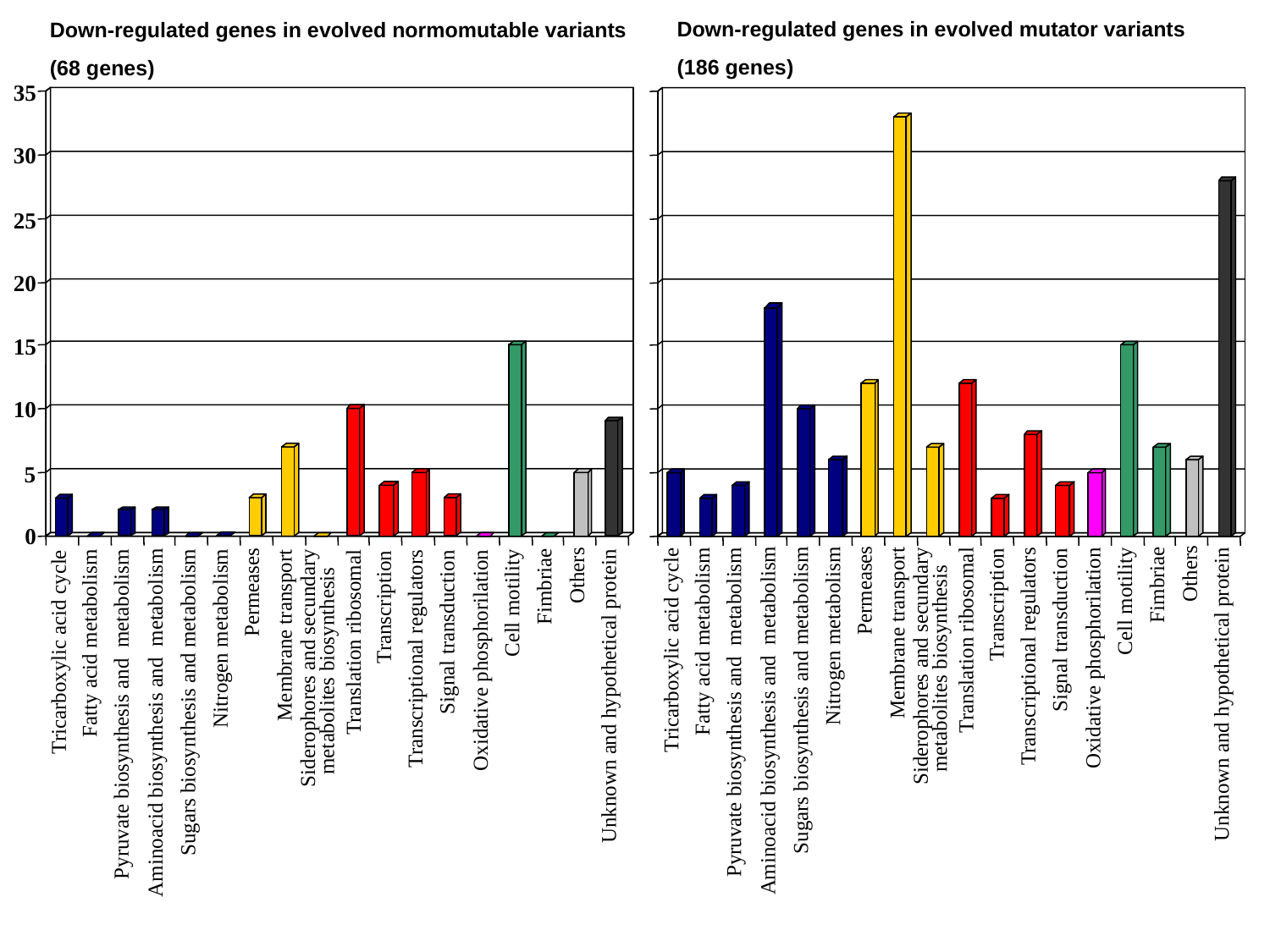

Down-regulated genes in evolved mutator variants
(186 genes)
Down-regulated genes in evolved normomutable variants
(68 genes)
35
30
25
20
15
10
5
0
Others
Fimbriae
Permeases
Cell motility
Transcription
Signal transduction
Membrane transport
Nitrogen metabolism
Translation ribosomal
Fatty acid metabolism
Tricarboxylic acid cycle
Transcriptional regulators
Oxidative phosphorilation
Unknown and hypothetical protein
Sugars biosynthesis and metabolism
Pyruvate biosynthesis and metabolism
Aminoacid biosynthesis and metabolism
Siderophores and secundary metabolites biosynthesis
Others
Fimbriae
Permeases
Cell motility
Transcription
Signal transduction
Membrane transport
Nitrogen metabolism
Translation ribosomal
Fatty acid metabolism
Tricarboxylic acid cycle
Transcriptional regulators
Oxidative phosphorilation
Unknown and hypothetical protein
Sugars biosynthesis and metabolism
Pyruvate biosynthesis and metabolism
Aminoacid biosynthesis and metabolism
Siderophores and secundary metabolites biosynthesis
