# Supplementary material for: Normal Mutation Rate Variants Arise in a Mutator (Mut S) Escherichia coli Population
Source: PLoS One. 2013 Sep 12;8(9):e72963. doi: 10.1371/journal.pone.0072963 (PMC3771984; doi:10.1371/journal.pone.0072963)
Supplement: Figure S9 — Distribution of the proportion of colonies with high and low mutation frequencies along four successive periods. (A) At the top, distribution of f values in 20 colonies of the ancestor strain; below, distributions of f values in 45 colonies from 15 different passages belonging to each one of the 1st (from 13th to 53rd passage), 2nd (from 58th to 93rd passage), 3rd (from 97th to 138th passage) and 4th (from 141st to 180th passage) periods respectively (see text S7 in File S2, is the same as Figure 3; it was reiterated here to facilitate comparisons). (B) Distribution of mutation frequencies in 20 colonies isolated from the same tube at particular passages along the evolution experiment. Black triangles and grey circles correspond to colonies with high- or low frequency of mutation respectively. (C) Distribution of mutation frequencies in 20 clones derived from single colonies with the highest and lowest f values but co-existing in particular passages (13th, 72nd, 129th and 180th). Black triangles and grey circles correspond to colonies with high- or low frequencies of mutation respectively. (PPT) [file pone.0072963.s009.ppt]

## Slide 1
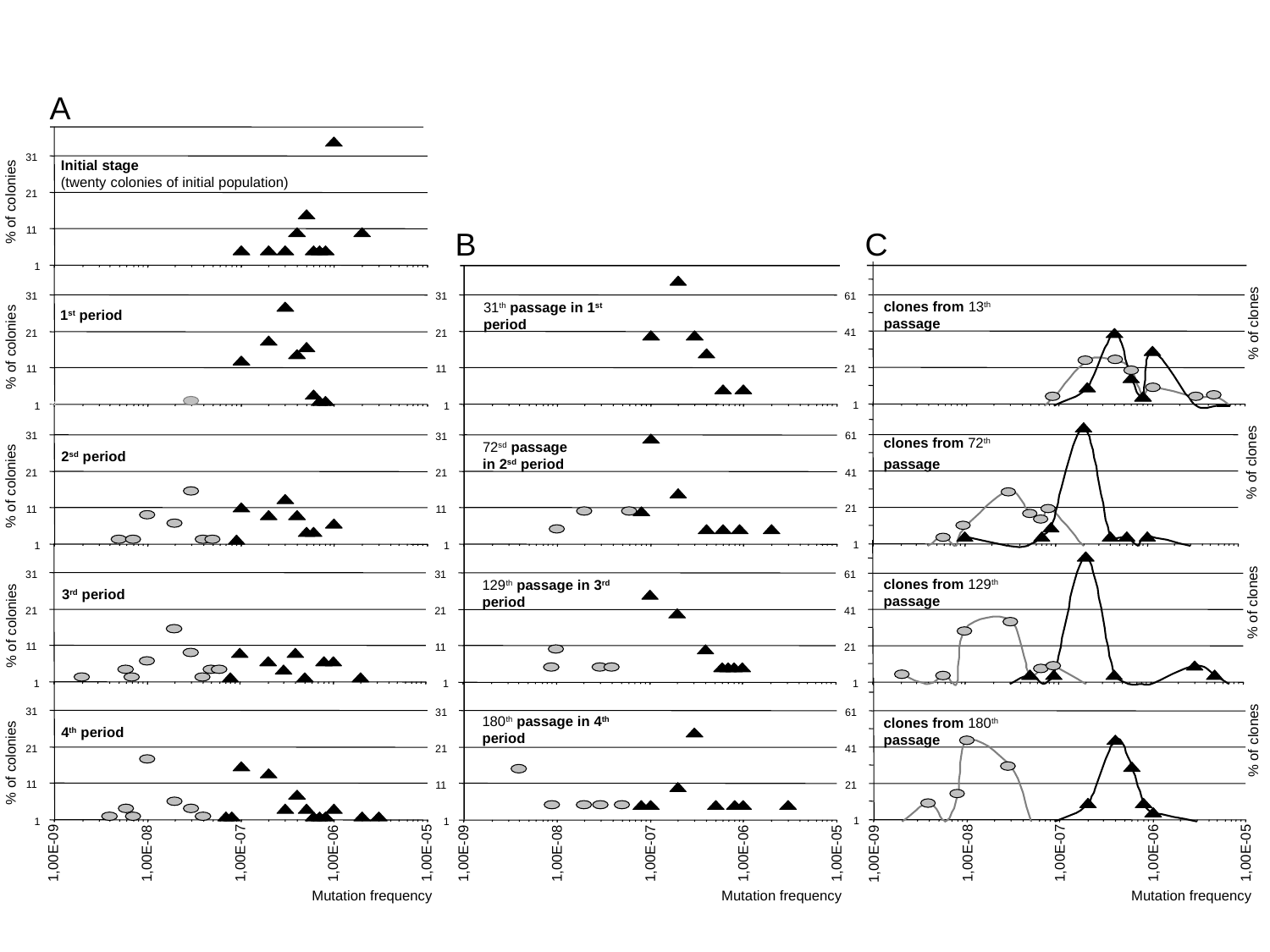

A
Initial stage
(twenty colonies of initial population)
31
21
11
1
31
1st period
21
11
1
1,00E-09
31
2sd period
21
11
1
31
3rd period
21
11
1
31
4th period
21
11
1
1,00E-09
1,00E-07
1,00E-05
1,00E-08
1,00E-06
% of colonies
C
clones from 13th passage
% of clones
clones from 72th passage
% of clones
clones from 129th passage
% of clones
clones from 180th passage
% of clones
B
31
31th passage in 1st period
21
11
1
31
72sd passage
in 2sd period
21
11
1
31
129th passage in 3rd period
21
11
1
31
180th passage in 4th period
21
11
1
1,00E-09
1,00E-08
1,00E-07
1,00E-06
1,00E-05
61
41
21
1
61
41
21
1
61
41
21
1
61
41
21
1
1,00E-08
1,00E-07
1,00E-06
1,00E-05
1,00E-09
% of colonies
% of colonies
% of colonies
% of colonies
Mutation frequency
Mutation frequency
Mutation frequency
