# Supplementary material for: Normal Mutation Rate Variants Arise in a Mutator (Mut S) Escherichia coli Population
Source: PLoS One. 2013 Sep 12;8(9):e72963. doi: 10.1371/journal.pone.0072963 (PMC3771984; doi:10.1371/journal.pone.0072963)
Supplement: File S1 — Supporting text. (DOC) [file pone.0072963.s010.doc]

**Text- Supporting Information 1**

**Normal Mutation Rate Variants Arise in a Mutator (Mut S) *Escherichia coli* Population**

**María-Carmen Turrientes, Fernando Baquero, Bruce R. Levin, José-Luis Martínez, Aida Ripoll, José-María M. González-Alba, Raquel Tobes, Marina Manrique, María-Rosario Baquero, Mario-José Rodríguez-Domínguez, Rafael Cantón, Juan-Carlos Galán.**

In the Introduction to this report, we used a verbal argument in support of the proposition that in the course of an experimental evolution study, it unlikely that strains with lower mutation rates will emerge in populations initially dominated by strains with high rates of mutation rates. Central to this argument is the assumption that if the only fitness cost of an elevated mutation rate is a higher rate of the generation of deleterious mutations, the cost of a high mutation rate would be small. In the extreme example considered, we assumed that the likelihood of generating a lethal mutation by a mutator strain is 1000 times greater than that of an otherwise isogenic strain with a normal mutation rate, say 10-3 and 10-6 per cell per generation, respectively. As a result the fitness cost of the non-mutator population, the selection coefficient, s would be on the order of ~0.001.

How long it will take for a low rate of mutation will be perceived can be calculated from classical population genetic theory [1]. If the initial relative frequencies of the low mutation and high mutation rate populations, M and N respectively are p and the q (p+q=1), the rate of increase in the frequency N would be

With a little calculation it can be show that the amount of time, t (generations) for the frequency of p to increase from some initial value p0 to some higher value t would be.

For example if p0=10-5 for the N population to reach a frequency of 10% pt=0.1, it would take approximately 9,315 generations.

Not considered in the above calculation are the effect of recurrent mutation from the high to low mutation rate populations, period selection and population wide bottlenecks. To consider the role of these processes the ascent of the non-mutator, we use semi-stochastic simulations for populations maintained at steady state, as in a chemostat. In these simulations, the changes in population density and selection are deterministic (differential equations solved by a finite step size Euler method) whilst the generation of mutants for the periodic selection simulation and bottlenecks are stochastic and simulated by a Monte Carlo process.

For the deterministic part of these simulations, N and M are the densities of the low and high mutation rate populations. The rate of growth of the populations is proportional to the concentration of a limiting resource, R µg/ml

$$\psi_{x}(R)=V_{x}\frac{R}{(R+k)}$$

where x is the designation of the strain and k, the concentration of the resource at which growth is half its maximum rate, V­x/2, [2, 3]. For these simulations we assume a chemostat like environment of unit volume, where C is the concentration of the resource in the reservoir and w per hour is the rate of flow of nutrients into the habitat (vessel). The rate of flow of excess resource out of the vessel as well as that of wastes and cells are is also equal to w.

With these definitions and assumptions in the basic model the rates of change in the densities of the N and M populations and change in the concentration of resource are given by

$$\begin{matrix} \frac{dR}{dt}=w(C-R)-e(\psi_{N}(R)N+\psi_{M}(R)M) \\ \frac{dN}{dt}=\psi_{N}(R)N-wN \\ \frac{dM}{dt}=\psi_{M}(R)M-wM \\ \end{matrix}$$

where *e*, the conversion efficiency is the amount or resource needed to produce a new cell [3].

**Periodic Selection:**

To simulate periodic selection, we assume that both the N and M populations generate higher fitness mutants at rates, probabilities per cell per hour, of µN and µM respective. Thus at each finite time step Δt there is a probability NµNΔt and MµMΔt that a single mutant of the next higher fitness state will be generated. If the random number x (0 ≤ x ≤ 1) is less than these probabilities, single cells of the next higher fitness state are produced and a single cell is removed from the generating population. A similar procedure was used for the generation of N mutants from the N population. In the simulations runs there are five fitness states with higher fitness mutants generated from the preceding lower fitness state. The Berkeley MadonnaTM program used for this simulation can be obtained from [www.eclf.net/programs](http://www.eclf.net/programs).

In Figure S1 (A) we present the results of a periodic selection simulation for a population initially dominated by lower fitness M cells (109 per ml and minority, 102 per ml higher fitness N cells. During the initial phase of this simulation, the N and M populations are sequentially replaced by higher fitness cells of their respective types. By the 1500th hour (about 425 generations) the highest fit state, 5, already dominates the community and all of the lower fitness earlier lineages are eliminated. Following the termination of periodic selection, the highest fitness, N5 population continues to increase in density. Since its fitness advantage is only 0.1% (s=0.001), the rate of its ascent is low.

In Figure S1 (B), we follow the changes in the densities of the total M and N populations. In the absence of periodic selection (line 1) the density of the N population increases monotonically. As a consequence of periodic selection, the there is a drop in the density of the ascending N population (line 2), but following the termination of this sequential adaptation to the cultures, the density of the N population ascends monotonically. In the absence of mutation from M to N (line 3), there is a substantial drop in the density of N during the periodic selection phase. But after the population is adapted, there are no longer higher fitness states, the frequency of the N population increases monotonically. With an increase in the rate of mutation from M to N, there is an increase in the density of the N population.

**Bottlenecks**

To illustrate the effect of bottlenecks on the dynamics of ascent of a rare higher fitness strain, we use a simulation in which there is a probability, pb per hour that the total population will crash and the density will be reduced by a factor β and then recover. In this simulation, mutation from N to M is deterministic but the bottlenecks are stochastic and simulated by a Monte Carlo routine similar to that used above. The program for this simulation is written in FORTRAN. The source code can be obtained from [www.eclf.net/programs](http://www.eclf.net/programs).

The bottlenecks are manifest by precipitous declines in then densities of the N and M populations followed by an increase in the concentration of the resource. With a low rate of recurrent N->M mutation (Figure S2 (A)), the rare N population is lost during the bottlenecks but recovers by mutation from the M. The recovered N cells may however be different from their ancestor N cells as they are arising *de novo*. However, as a consequence of the bottlenecks, in the run shown the frequency of the N population remained low and did not increase. Although the initial densities of the N population are below that which would survive a bottleneck, as a consequence of high rate of recurrent mutation from M to N, the N population is not lost as a consequence of the bottlenecks (Figure S2 (B)). Moreover, the frequency of this population is relatively higher than that with a low rate of mutation and continues to increase.

**REFERENCES**

1. Crow JF, Kimura M (1971) An Introduction to Population Genetics Theory (Harper Row, New York) First Ed p 591.
2. Monod J (1949) The growth of bacterial cultures. Annu Rev Microbiol 3:371-394.
3. Stewart FM, Levin BR (1973) Partitioning of resources and the outcome of interspecific competition: a model and some general considerations. Am Nat 107:171-198.
