# Supplementary material for: Normal Mutation Rate Variants Arise in a Mutator (Mut S) Escherichia coli Population
Source: PLoS One. 2013 Sep 12;8(9):e72963. doi: 10.1371/journal.pone.0072963 (PMC3771984; doi:10.1371/journal.pone.0072963)
Supplement: File S2 — Contains supporting text and Table S1 and Table S2. Table S1. Complementation of the ancestor strain with genes commonly involved in the mutator phenotype. Table S2. Mutation rates of the Escherichia coli ECU24 original strain (t0), an evolved normo-mutable variant and an evolved mutator variant (both from t151 passage) over-expressing Nei, SodB, and KatG. (DOC) [file pone.0072963.s011.doc]

**Text- Supporting Information 2**

**Normal Mutation Rate Variants Arise in a Mutator (Mut S) *Escherichia coli* Population**

**María-Carmen Turrientes, Fernando Baquero, Bruce R. Levin, José-Luis Martínez, Aida Ripoll, José-María M. González-Alba, Raquel Tobes, Marina Manrique, María-Rosario Baquero, Mario-José Rodríguez-Domínguez, Rafael Cantón, Juan-Carlos Galán.**

**S1. Complementation of the ancestor strain with genes commonly involved in the mutator phenotype.** The genes more commonly involved in mutator phenotype (*mutS, mutL, mutH, uvrD, mutT* and *mutY*) were amplified from the *E.coli* MG1655 strain using primers and PCR conditions previously described [1]. The clones containing the expected insert were twice re-checked and finally the inserts were sequenced to confirm the absence of mutations when compared with the wild-type strain. The hybrid plasmids carrying each one of the mentioned genes were transformed into the original mutator strain. Mutation frequencies (*f*) were determined for all strains carrying these constructions, according to a previously described protocol [*2*]. *f* was obtained by determining the proportion of rifampin-resistant colonies per total viable cell count. The results are shown in Table S1.

The original mutator strain transformed with wild-type *mutS* gene reverted to low mutation frequency phenotype. A modest reduction in *f* was observed when *mutL* and *mutH* genes were introduced in the original mutator strain. This effect has been previously described in both mutator and non-mutator *E.coli* strains [2, 3]. The small decrease in *f* observed when *uvrD* and *mutT* were over-expressed has not been previously reported. We sequenced the *uvrD* and *mutT* genes in the original strain. No amino acid changes were found with respect to the corresponding sequence in *E.coli* MG1655.

**S2. Emergence of normo-mutable variants in six replicate experiments**. Colonies with *f* values compatible with a normo-mutable phenotype were detected in all six bacterial replicate populations derived from the same ancestral population, ECU24-t_0_, and submitted in parallel to 80 serial passages.

The *f* for three independent colonies of each replicate was estimated in the ancestor cultures, in the first perio*d* (13^th^ passage) and in the *second* period (72^nd^ passage). Pooled mutation frequencies of colonies from original clinical strain cultures ranged from 3x10^-6^ to 4x10^-7^, and *f* values ranged similarly in colonies of the first period (13^th^ passage), from 3x10^-6^ to 1x10^-7^. However, in the *second* period (72^nd^ passage) *f* values were ranging from 1x10^-6^ to 1x10^-8^, with an important broadening (100-fold) in the range of mutation frequencies, due to the emergence of normomutable variants. At this passage, 17% of the colonies entered into the normo-mutable range of mutation frequencies (*f*= 6.38x10^-8^ to 5.95x10^-9^) a very similar proportion to that detected in the main experiment. PFGE experiments confirmed the identity of the ECU24 strain along the serial passages (Figure S3).

**S3. Fitness of mutators and normo-mutable variants**

Fitness of normo-mutable and mutator colonies obtained along the experiment was measured as relative growth rates (σ) in relation to original mutator clinical strain (arbitrary assigned as σ=1.00). During the first ~400 generations, while the mutator population remained essentially homogeneous in terms of mutation rate, fitness was increased, reaching a mean σ=1.69 (Figure S4). This increase reflects the acquisition by the original mutator strain of adaptive changes optimizing its fitness in the new environment (from urine to broth). In successive periods, when the mutator population started to give rise to normo-mutable variants, the mean σ values of the mutator-derived colonies were maintained (slightly reduced?). Their fitness values reduced from the optimum 1.69 to 1.46 and 1.54 in the 2^nd^, and 3^rd^ periods, recovering the level of the ancestral strain (0.96) in the 4^th^ period. It is of note that even at the 4^th^ period, a number of mutator colonies retained fitness values close to the highest ones (σ=1.69). That suggests that accumulated mutations did not necessarily affect fitness, or more probably that mutators are also endowed to increase the possibility of finding compensatory mutations restoring the cost of deleterious mutations. Normo-mutable derived colonies had mean fitness values of σ=1.48, 1.69 and 1.60 at the 2^nd^, 3^rd^, and 4^th^ periods respectively, higher than mutators particularly during the last periods.

**S4. Changes in gene expression in evolved mutators and normomutable variants and with respect to the original mutator strain.** The full genome RNA expression level of 4,494 genes of *E.coli* MG1655 was compared between the ancestral strain and three evolved normo-mutable variants and three evolved mutators. All genes showing statistically significant differences in the expression level were selected following the AffymetrixGenechip® technology manufacturer’s instructions. Only genes whose up-regulation was consistently present in replicates from the three colonies belonging to the same group (normo-mutable or mutator) are shown ([http://bioinfogp.cnb.csic.es/tools/venny/index.html](http://bioinfogp.cnb.csic.es/ tools/venny/index.html)). Among up-regulated genes, those involved in amino acid biosynthesis and metabolism were mainly observed in the mutator strains, whereas genes belonging to glyoxalate and pyruvate biosynthesis, involved in tricarboxylic acid cycle, or in fatty acid metabolism were over-represented among normo-mutable colonies. As in up-regulated genes, only genes whose down-regulation was consistently present in replicates from the three colonies belonging to the same group (normo-mutable or mutator) are shown (Figure S5). Among down-regulated genes, those related to membrane transport and production of siderophores, were more frequently found in the mutator colonies (Figure S6).

Among the 94 genes exclusively up-regulated in the normo-mutable variant strain, 22 genes were involved in TCA cycle and acetate consumption, suggesting that this strain consumes acetate and display a high TCA cycle activity. On the other hand, genes involved in acetate efflux (as *pflB*, *pta* and *ackA*) were over-expressed in the mutator strain. These results strongly suggest an acetate cross-feeding process between normo-mutable and mutator cells contributing to long-standing coexistence of both of them. Besides this potential cross-feeding based on acetate excretion and consumption in exhausted LB, we cannot exclude that other nutritional trade-offs might occur in other variants of the same population.

**S5. Mutation rates of the *Escherichia coli* ECU24 original strain (t_0_) a normo-mutable variant and an evolved mutator (both from t_151_ passage) over-expressing KatB, SodB, and Nei.**

Reduction in mutation rates due to the hyper-expression of anti-superoxide genes was higher in the original mutator strain, suggesting that both normo-mutable variants and evolved mutators already have a certain degree of hyper-expression of these genes. Note that the evolved mutator strain obtained from a late passage (t_151_) has a 5-times lower mutation rate than the original strain (see Table S2).

**S6. Fitness of ancestor variants over-expressing SodB, Nei, and KatG.**

In the previous paragraph it was shown that over-expression of Nei, SodB, and KatG reduced the high mutation rates of the ancestor strain. The same strains were used to perform fitness experiments based on determination of relative growth rates (see Text S3). The highest reductions in mutation rates were obtained with the over-expression of SodB in the ancestor strain (9.27x reduction). Significantly, the RGR average of 15 repeated measures from colonies derived from three ancestor clones over-expressing SodB was 1.35, that is, the growth rate of the ancestor mutator strain increased by 35%. Consistently with the low effect of the over-expression of SodB in reducing mutation rates in evolved variants, average RGR values of both late (t_151_) variants over-expressing SodB were not modified (RGR 0.97 and 0.99). The over-expression of Nei in the ancestor clone yielded an average RGR of 1.05, but one of the clones had RGR=1.26, also indicating a fitness increase related with Nei over-expression. Again, this fitness gain was not detectable in evolved (t_151_) variants, showing reductions in RGRs ranging from 0.84-0.97. The over-expression of KatG in the three independent clones derived from the ancestor clone produced an average increased RGR of 1.05, but one of the clones reached RGR= 1.22. Consistently with the above results for other superoxide genes, no fitness gain was observed in evolved variants, with reductions in RGRs ranging 0.81-0.93. In summary, over-expression of superoxide scavenging genes increased bacterial fitness of the original mutator strain, particularly with SodB, but such gain is not observed in late variants in which over-expression is already present (see Figure S4).

**S7. Coexistence of mutator and normo-mutable cells in the same culture.**

As it will be shown later (see Text S8 and Figures S7 and S8), genomic data demonstrates the coexistence of different variants in the same culture. In order to confirm the coexistence of two distinct populations without overlapping *f* values, mutation frequencies were estimated for 20 independent colonies obtained from a particular passage (the 31^st^) from the first period (late before diversification) and 20 colonies from particular passages of the second (72^nd^), third (129^th^) and fourth (180^th^) periods. According to *f* values obtained, all colonies from the first period passage corresponded to the mutator population, whereas colonies belonging to either the mutator (f> 7.00x10^-8^) or normo-mutable populations (f< 7.00x10^-8^) were consistently found in coexistence during the subsequent periods (Figure S9A). Twenty clones derived from single colonies representing extreme values in the *f* distribution (highest and lowest of Figure S9B) for four particular passages along the experiment were analyzed (Figure S9C). In the first period (13^th^ passage) the *f* clonal distributions derived from the colonies with the highest and lowest *f* could not be differentiated, suggesting the homogeneity of the population. Two populations with different mutational phenotypes were progressively differentiated in the successive periods (72^nd^, 129^th^ passages). Finally, distinct peaks for mutators and non-mutators illustrate their coexistence in the 4^th^ period, 180^th^ passage, (p<0.001) (Figure S9C).

**S8. Genome alignment of the two normo-mutable and two mutator strains coexisting at t_151_ and t_180_ cultures against the ancestor strain t_0_**. The genomes of t_151_ and t_180_ normo-mutable strains, were aligned with progressive MAUVE against the ancestor strain t_0_. The genomes show large blocks of similarity with some insertions/deletions of small fragments (Figures S7 and S8). Some transposition events can be observed as expected considering the high frequency of transposases. Most of the deletions/insertions are in phage regions and in plasmid contigs or are related with different allocations of the transposases active in each genome. Based on MAUVE alignment results the insertions, deletions and SNPs were exhaustively analysed along the genome. Results indicate the possibility of coexistence of different *E. coli* ECU24-derived strains in the same culture tube.

**S9. Competition experiments in late coexisting mutator and normo-mutator clones.**

Spontaneous derivatives able to metabolize arabinose (ara ^+^) were isolated from both hyper-mutable and normo-mutable populations that were present at the t_180_ passage of the wild-type evolved *ara* – *E. coli* ECU24 by plating in arabinose-McConkey agar plates. The stability of the variants was ascertained in six repeated passages. Mutator and normomutable t_180_ ara – and ara ^+^ strains were streaked onto agar plates for single colonies. Colonies were then inoculated into LB broth and incubated overnight. At time zero (t_0_) of the competition experiment, 50 μl of the two competitors (one of which would be ara – and the other ara ^+^) were mixed in the same proportion into 20 ml flasks of fresh LB broth. A sample of this mixed competition culture (and suitable controls) was immediately taken, appropriately diluted, and spread onto McConkey agar base (Becton Dickinson, Sparks, US) plates with arabinose (1%) The mixed broth culture was incubated for 24 h at 37ºC with shaking (200 rpm), then another 50 µl sample was seeded in fresh 20 ml LB broth flasks (1:400). The relative densities of each competitor after 24 h of competition were evaluated by counting red or white colonies after sampling on McConkey agar plates. Competition was followed during four days. Five independent replicate competition experiments were performed. The relative fitness (W) was estimated as described by Travisano and Lenski [4]. Every experiment was done in a direct and an inverse way, to evaluate the relative cost of bacterial hosts and plasmids.

When strains with the ara+ phenotype were competing with those of the ara- phenotype, a small but constant cost of strains harbouring the ara+ phenotype (0.02 per generation/day) was detected, which might be attributed to the known methylglyoxal toxicity that follows hyper-induction of *araBAD* [5]*.* This observed cost was identical (relative fitness W = 1) regardless of whether the ara+ variant had the mutator or normo-mutable phenotypes, in competition with the ara- normomutable or mutator ones, indicating the short-term neutrality of late mutational phenotypes.

**REFERENCES**

1. Galán JC, Turrientes MC, Baquero MR, Rodríguez-Alcayna M, Martínez-Amado J, et al. (2007) Mutation rate is reduced by increased dosage of *mutL* gene in *Escherichia coli* K-12. FEMS Microbiol Lett 275(2): 263-269.
2. Baquero MR*,* Nilsson AI, Turrientes MC, Sandvang D, Galán JC, et al. (2004) Polymorphic mutation frequencies in *Escherichia coli*: emergence of weak mutators in clinical isolates. J Bacteriol 186(16): 5538-5542.
3. Schaaper RM, Radman M (1989) The extreme mutator effect of *Escherichia coli mutD*5 results from saturation of mismatch repair by excessive DNA replication errors. EMBO J 8(11): 3511-3516.
4. Travisano M, Lenski RE (1996) Long-term experimental evolution in *Escherichia coli.* IV. Targets of selection and the specificity of adaptation. Genetics 143:15-26.
5. Bankaitis VA, Kline EL (1981) Cyclic adenosine 3´,5´-monophosphate-mediated hyperinduction of araBAD and lacZYA expression in a crp mutant of *Escherichia coli* K12. J Bacteriol 147: 500-508.

**Supporting INFORMATION 1 TABLES**

**Table S1. Complementation of the ancestor strain with genes commonly involved in the mutator phenotype.**

| **Strain** | **Mutation Frequency** | **Reduction**  **(fold)** |
| --- | --- | --- |
| t_0_ wild-type | 160.0×10^-8^ |  |
| **t_0_ (pGEMt-*mutS*)** | **2.53×10^-8^** | **63.24** |
| t_0_ (pGEMt-*mutL*) | 29.2×10^-8^ | 5.47 |
| t_0_ (pGEMt-*mutH*) | 32.9×10^-8^ | 4.86 |
| t_0_ (pGEMt-*uvrD*) | 52.3×10^-8^ | 3.05 |
| t_0_ (pGEM-*mutT*) | 42.3×10^-8^ | 3.78 |
| t_0_ (pGEMt-*mutY*) | 142.0 ×10^-8^ | 1.12 |

**Table S2. Mutation rates of the *Escherichia coli* ECU24 original strain (t_0_), an evolved normo-mutable variant and an evolved mutator variant (both from t_151_ passage) over-expressing Nei, SodB, and KatG.**

| **Strain** | **Mutation rate** | **Confidence intervals** | **Reduction (fold)** |
| --- | --- | --- | --- |
| **Original mutator t_0_** | 18.7×10^-8^ | (14.2-21.0) ×10^-8^ |  |
| (pGEMt-*nei*) | 4.26×10^-8^ | (2.90-5.78) ×10^-8^ | **4.39** |
| (pGEMt-*sodB*) | 2.02×10^-8^ | (1.43-2.68) ×10^-8^ | **9.27** |
| (pGEMt-*katG*) | 4.98 x10^-8^ | (4.01-6.04) x 10^-8^ | **3.76** |
| **Normo-mutable** | 0.28×10^-8^ | (0.21-0.36) ×10^-8^ |  |
| (pGEMt-*nei*) | ^1^ND | - |  |
| (pGEMt-*sodB*) | 0.26×10^-8^ | (0.19-0.34) ×10^-8^ | 1.09 |
| (pGEMt-*katG*) | 0.26×10^-8^ | (0.19-0.34) ×10^-8^ | 1.09 |
| **Evolved Mutator** | 3.41×10^-8^ | (2.49-4.42) ×10^-8^ |  |
| (pGEMt-*nei*) | 4.99×10^-8^ | (3.47-6.72) ×10^-8^ | 0.68 |
| (pGEMt-*sodB*) | 1.40×10^-8^ | (1.02-1.82) ×10^-8^ | 2.44 |
| (pGEMt-*katG*) | 6.59×10^-8^ | (5.30-7.96) ×10^-8^ | 0.51 |

^1^ND.- Not determined.
